# Dose-resolved control of somatic reprogramming by *Rora*

**DOI:** 10.1016/j.stemcr.2026.102870

**Published:** 2026-04-02

**Authors:** Haiyun Wang, Yusha Li, Chunkou Yin, Zhen Zhang, Yixuan Wang, Yi Li, Chuang Li, Runxia Lin, Xiaoli Zhang, Jing Guo, Rongping Luo, Shumin Li, Lv Zhang, Yingting Zhuang, Anchun Xu, Jiani Wan, Lizhan Xiao, Bailing Chen, Shengyong Yu, Manish Kumar, Jing Liu

**Affiliations:** 1 China-New Zealand Joint Laboratory on Biomedicine and Health, Guangdong Provincial Key Laboratory of Stem Cell and Regenerative Medicine, Guangdong-Hong Kong Joint Laboratory for Stem Cell and Regenerative Medicine, Institute of Development and Regeneration, Guangzhou Institutes of Biomedicine and Health, Chinese Academy of Sciences, Guangzhou 510530, China; 2 Centre for Regenerative Medicine and Health, Hong Kong Institute of Science & Innovation, Chinese Academy of Sciences, Hong Kong SAR, China; 3 Joint School of Life Sciences, Guangzhou Institutes of Biomedicine and Health, Chinese Academy of Sciences, Guangzhou Medical University, Guangzhou 510530, China; 4 University of Chinese Academy of Sciences, Beijing 100049, China; 5 School of Life Sciences, University of Science and Technology of China, Hefei 230027, China; 6 School of Pharmacy, Fujian Medical University, Fuzhou 350122, P.R. China; 7 Institutes of Biomedical Sciences, Shanxi Provincial Key Laboratory for Medical Molecular Cell Biology, Key Laboratory of Chemical Biology and Molecular Engineering of Ministry of Education, Shanxi University, Taiyuan 030006, China

**Keywords:** Rora, nuclear receptors, somatic cell reprogramming, dose-dependent regulation

## Abstract

Nuclear receptors (NRs) are ligand-regulated transcription factors whose domains and dosage modulate gene networks. We systematically profiled 49 murine NRs in OKS (OCT4/KLF4/SOX2) reprogramming of mouse fibroblasts and identified the ROR subfamily as enhancers. Focusing on *Rora*, we uncovered a dose-dependent, biphasic effect on reprogramming: moderate *Rora* increases OCT4-GFP^+^ colony formation, whereas higher expression reduces colonies. Domain dissection separated these arms—DNA-binding domain (DBD)/ligand-binding domain (LBD) was required for the pro-reprogramming effect, while the N-terminal domain (NTD) was required for high-dose inhibition (ΔNTD eliminated the inhibitory limb). Functionally, the reprogramming barrier interferon (IFN)-γ was attenuated at transcript and protein levels; IFN-γ add-back dampened the enhancement, supporting immune-axis modulation. Conversely, WNT pathway output was reduced in the inhibitory arm, and CHIR99021 partially rescued the high-dose colony defect. Thus, RORA acts as a dose-programmed, domain-modular regulator that coordinates chromatin and signaling to gate cell-fate conversion, establishing nuclear-receptor dosage control as a lever to improve reprogramming efficiency.

## Highlights

• Systematic screen of 49 murine nuclear receptors identifies RORs as OKS enhancers
• RORA exerts a dose-dependent, biphasic effect on OCT4-GFP^+^ colony formation
• Pro-reprogramming requires DBD/LBD; high-dose inhibition requires NTD
• Rora^Low^ limits IFN-γ; add-back blocks benefit. High lowers WNT; CHIR partly rescues

## Graphical abstract

**Figure:**
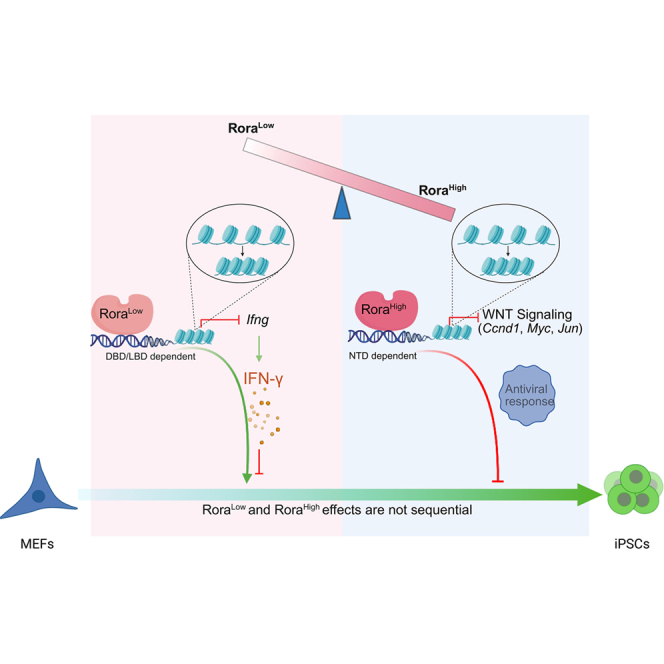

## Teaser

Liu and colleagues report that *Rora*’s impact on reprogramming is dose sensitive: DBD/LBD-dependent low dose enhances reprogramming in part through limiting IFN-γ signaling, whereas NTD-dependent high dose inhibits reprogramming with reduced WNT pathway output and weakening antiviral programs.

## Introduction

Direct reprogramming of somatic cells to induced pluripotent stem cells (iPSCs) by transcriptional factors resets cell identity but remains constrained by cell-intrinsic barriers and incomplete control of chromatin and signaling (Chen et al., 2011; Takahashi et al., 2007; Takahashi and Yamanaka, 2006; Wang et al., 2019). Nuclear receptors (NRs), a superfamily of ligand-regulated transcription factors with modular N-terminal domain (NTD), DNA-binding domain (DBD), and ligand-binding domain (LBD), recruit chromatin cofactors to regulate broad gene networks governing development, metabolism, immunity, circadian rhythms, and proliferation (Choi et al., 2023; Frigo et al., 2021; Mazaira et al., 2018; Weikum et al., 2018). Since the first NRs were cloned, 49 murine NRs have been cataloged (Bookout et al., 2006; Sonoda et al., 2008; Xie et al., 2009; Yang et al., 2006). Dysregulated NR activity contributes to diverse diseases, while precisely targeted ligands can reprogram gene expression, making NRs compelling candidates for improving reprogramming efficiency and fidelity (Choi et al., 2023; Frigo et al., 2021; Mazaira et al., 2018; Weikum et al., 2018).

Multiple NRs intersect pluripotency and reprogramming. NR5A2 (LRH-1) can replace OCT4 and enhance OKSM reprogramming (Gonzales and Ng, 2013; Heng et al., 2010; Kobayashi et al., 2024); ESRRB reinforces pluripotency circuitry and activates *Oct4* (Feng et al., 2009; Martello et al., 2012). Retinoid pathways are similarly influential: RARγ and LRH-1 accelerate reprogramming in a stage-sensitive manner (Taei et al., 2020), and the RXR agonist CD3254 boosts chemical reprogramming by activating endogenous RXRα (Jin et al., 2023). These studies suggest that NR dosage, domain architecture, and ligand timing could together determine reprogramming outcomes, yet a systematic, mechanistic view has been lacking.

The retinoic acid receptor-related orphan receptors (RORs; NR1F: RORA, RORB, RORC) bind the ROR response element (RORE) and coordinate circadian, developmental, metabolic, and immune programs (Cook et al., 2015; Jetten, 2009; Ma et al., 2021; Zhang et al., 2023). Two pathway axes make RORs especially compelling in reprogramming. First, interferon-γ (IFN-γ) and broader IFN programs can constitute barriers that reduce iPSC yield (Guo et al., 2019; Ivashkiv, 2018; Qiao et al., 2013). Second, WNT/β-catenin signaling exerts stage- and dose-dependent effects, dampening early acquisition yet promoting later stabilization of pluripotency, with small-molecule activation (e.g., CHIR99021) commonly used to modulate outcomes (Grainger and Willert, 2018; Ho et al., 2013; Kim et al., 2020; Zhang et al., 2014). These features raise a specific mechanistic question: can RORs tune OKS reprogramming by coordinating chromatin accessibility with immune and WNT outputs in a dose- and domain-dependent manner?

Here, we systematically profiled mouse NRs in OKS reprogramming and identified the ROR subfamily as a coherent enhancer group. Focusing on *Rora*, we uncovered a biphasic dose response: Rora^Low^ increases OCT4-GFP^+^ colony numbers, whereas Rora^High^ decreases colony formation (here, Rora^Low^ and Rora^High^ denote low and high viral input (dosage) of *Rora*, respectively. Rora^ΔNTD^, Rora^ΔDBD^, and Rora^ΔLBD^ denote domain-deletion constructs). Domain dissection showed that the DBD and LBD are required for the pro-reprogramming arm, while the NTD is required for high-dose inhibition. CUT&Tag and ATAC-seq demonstrated RORA binding at RORE-anchored elements across conditions, with dose-dependent co-motif shifts and accessibility changes. Transcriptome and functional assays linked Rora^Low^ to attenuation of IFN-γ signaling and Rora^High^ to reduced WNT pathway output, the latter rescued by CHIR99021. Collectively, these data define a dose- and domain-resolved mechanism by which RORA coordinates chromatin and signaling to gate cell-fate conversion and they highlight nuclear-receptor dosage control as a practical axis for optimizing reprogramming.

## Results

### Screen of nuclear receptors identifies the ROR subfamily as coherent enhancers of OKS-mediated reprogramming

**Figure 1:**
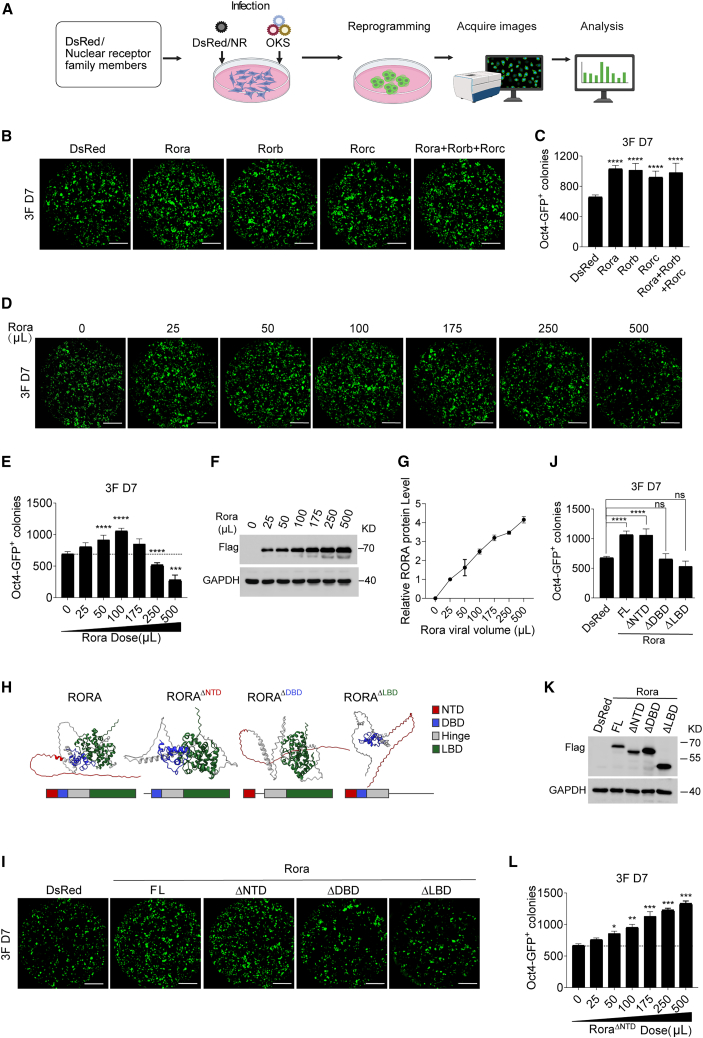
*Rora* exerts dose-dependent effects on OKS-mediated reprogramming (A) Schematic of the screening strategy evaluating 49 NR family members in an OKS reprogramming system using MEFs. (B) Effects of the ROR subfamily on OKS reprogramming. Representative whole-well scans of OCT4-GFP^+^ colonies on D7 from 1 × 10^4^ MEFs transduced with dsRed (control), *Rora*, *Rorb*, *Rorc*, or the combination *Rora* + *Rorb* + *Rorc* in iCD1 medium. Scale bars, 5 mm. (C) Quantification of OCT4-GFP^+^ colony numbers corresponding to (B). Data are mean ± SD; one-way ANOVA, Dunnett’s test; *n* = 3 independent experiments; ^∗∗∗∗^*p* < 0.0001. (D) Representative whole-well scans illustrating the effect of graded *Rora* dosing on OKS reprogramming. Scale bars, 5 mm. (E) Quantification corresponding to (D), including the dsRed control. Data are mean ± SD; one-way ANOVA, Dunnett’s test; *n* = 3 independent experiments; ^∗∗∗^*p* < 0.001, ^∗∗∗∗^*p* < 0.0001. (F) Immunoblot of RORA protein levels on D3 for the *Rora* dose series used in (E). (G) Densitometric quantification of (F). (H) Domain architecture of RORA and deletion mutants: ΔNTD (N-terminal domain), ΔDBD (DNA-binding domain), and ΔLBD (ligand-binding domain). Protein structures were predicted using AlphaFold and visualized in UCSF ChimeraX with color formatting applied. (I) Representative whole-well scans showing the effects of *Rora* and domain-deletion mutants on OKS reprogramming relative to dsRed control (OCT4-GFP^+^ colony formation). Scale bars, 5 mm. (J) Quantification corresponding to (I). Data are mean ± SD; one-way ANOVA, Dunnett’s test; *n* = 3 independent experiments; ^∗∗∗∗^*p* < 0.0001; ns, not significant. (K) Immunoblot showing RORA expression on D3 for full-length (FL) and domain-deletion constructs used in (J). (L) Effects of graded dosing of Rora^ΔNTD^ on OKS reprogramming (OCT4-GFP^+^ colonies) relative to dsRed control. Data are mean ± SD; one-way ANOVA, Dunnett’s test; *n* = 3 independent experiments; ^∗^*p* < 0.05, ^∗∗^*p* < 0.01, ^∗∗∗^*p* < 0.001.

To systematically evaluate the roles of mouse NRs in somatic cell reprogramming, we cloned 49 NR family members and co-expressed each with OKS in mouse embryonic fibroblasts (MEFs), cultured in iCD1 induction medium (Chen et al., 2011). OCT4-GFP^+^ colonies were quantified on day (D) 7. Seven factors significantly increased reprogramming efficiency (*Rora*, *Rorb*, *Rorc*, *Nr1i3*, *Nr3c2*, *Nr5a1*, and *Nr5a2*), whereas 14 factors reduced colony formation (Figures 1A and S1A). Notably, the ROR subfamily (*Rora*/*Rorb*/*Rorc*) was the only family-level group in which all three members significantly increased OCT4-GFP^+^ colonies in the primary screen (Figure S1A); *Nr5a1* and *Nr5a2* served as positive references consistent with prior reports. The ROR subfamily shares a conserved zinc-finger DBD and recognizes the same monomeric RORE—an AT-rich 5′ extension preceding the AGGTCA core—while differing primarily in NTD/AF-1 length and sequence. RORs have well-established roles in immunity (e.g., RORγ in Th17 differentiation), circadian regulation (RORα/β in clock gene control), and development (e.g., RORα in cerebellar Purkinje cells) (Lee et al., 2021). Motivated by these functions, we asked whether their effects on reprogramming intersect immune or circadian programs; however, prior work directly linking RORs to somatic cell reprogramming is sparse.

### *Rora* exhibits a biphasic, dose-dependent effect on reprogramming efficiency

We first tested whether ROR family members cooperate to enhance OKS reprogramming. *Rora*, *Rorb*, *Rorc*, and the triple combination (*Rora* + *Rorb* + *Rorc*) were expressed individually in MEFs alongside OKS under otherwise identical conditions. No synergistic enhancement was observed for the triple combination beyond the best single-factor effect (Figures 1B and 1C). Because *Rora* produced the largest and most reproducible increase in OCT4-GFP^+^ colonies among the three, we prioritized *Rora* for mechanistic dissection in subsequent experiments.

Titration of *Rora* over a broad viral input range revealed a biphasic response of OCT4-GFP^+^ colony numbers: colonies increased with rising *Rora* dose up to a peak and then declined, ultimately falling below the dsRed control at the highest doses (Figures 1D–1G). We, therefore, define a dose-dependent promotion followed by inhibition at suprathreshold *Rora* levels.

To control for potential variability in viral delivery, we quantified co-infection efficiency at D2 (72 h after the second infection). *Rora* input produced a graded increase in FLAG-positive cells, whereas SOX2 positivity and exogenous OKS transcript/vector DNA levels were unchanged across the dose series, indicating that the dose-dependent phenotypes are not explained by altered OKS infectivity (Figures S1B–S1D).

### RORA domains and isoforms specify dose-dependent reprogramming: DBD/LBD drives promotion and NTD mediates high-dose inhibition

NRs share a conserved modular architecture comprising an NTD (AF-1), a DBD, a hinge region, and a C-terminal LBD (AF-2), with the greatest isoform-specific variability typically residing in the NTD (Jin et al., 2025). Consistent with this design, RORA contains an NTD, DBD, hinge, and LBD. Domain deletions demonstrated that the DBD and LBD are required for the pro-reprogramming activity, whereas the NTD is dispensable for promotion but necessary for high-dose inhibition: ΔDBD and ΔLBD abolished the enhancement, while ΔNTD preserved the enhancement and eliminated the inhibitory limb at high viral input (Figures 1H–1L). Because isoform diversity among ROR factors is concentrated in the NTD, we next asked whether dose dependence tracks with NTD length. Mouse *Rora* encodes two isoforms (*Rora1* and *Rora4*), as do *Rorb* (*Rorb1* and *Rorb2*) and *Rorc* (*Rorc1* and *Rorc2*). Among these, *Rora1* has the longest NTD. Consistent with the ΔNTD result, only *Rora1* showed a biphasic, dose-dependent response, whereas *Rora4*, *Rorb1/2*, and *Rorc1/2* behaved like ΔNTD-enhancing reprogramming without high-dose inhibition (Figures S2A and S2B).

Importantly, titration of the dsRed control virus across the same input range had no detectable effect on OCT4-GFP^+^ colony output (Figure S2C), arguing against a non-specific viral-load explanation for the high-dose inhibitory phenotype. In parallel, quantification of FLAG immunoblots showed that ΔDBD and ΔLBD constructs accumulated at modestly higher protein levels than full-length RORA under matched infection conditions (Figure S2D); nevertheless, these constructs did not display dose-dependent inhibition in the corresponding titration experiments (Figures S2E and S2F). Thus, the biphasic response is not explained by generalized viral burden or modest differences in protein abundance, but instead tracks with an NTD-dependent inhibitory mechanism uniquely engaged at suprathreshold full-length *Rora1* expression.

Together, these data support a model in which DBD/LBD-dependent transcriptional activity drives promotion, while an NTD-dependent mechanism uniquely confers dose-dependent inhibition at suprathreshold expression.

### *Rora* dosage reshapes transcriptional programs and protein networks during OKS-mediated reprogramming

**Figure 2:**
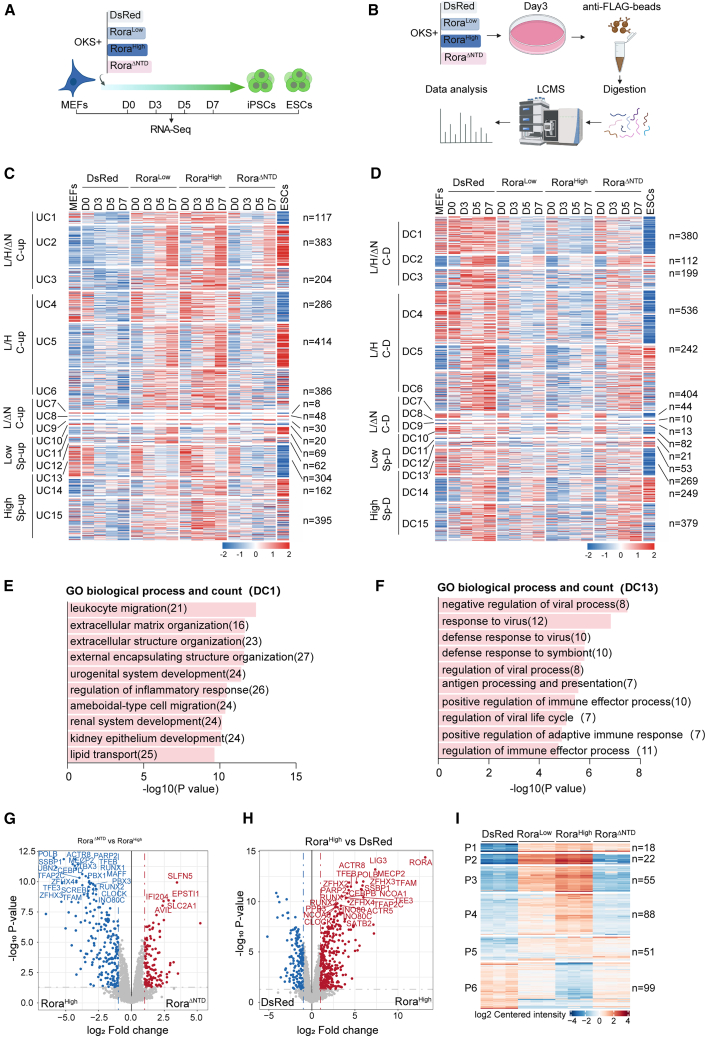
*Rora* dosage reshapes transcriptional programs and protein networks during OKS-mediated reprogramming (A) RNA-seq experimental design. MEFs were transduced with OKS together with dsRed (control), Rora^Low^, Rora^High^, or Rora^ΔNTD^ (N-terminal domain deletion). Samples were collected at D0, D3, D5 and D7; MEFs and ESCs were included as a reference. (B) IP-MS spectrometry workflow. D3 reprogramming cultures expressing dsRed, Rora^Low^, Rora^High^ or Rora^ΔNTD^ were subjected to FLAG-based IP followed by liquid chromatography (LC)-MS to profile proteins enriched with each condition (biological triplicates). (C) Heatmap of genes upregulated in Rora^Low^, Rora^High^ and Rora^ΔNTD^ relative to dsRed across the reprogramming time course. Genes were grouped into 15 Up-clusters (UC1-UC15) by hierarchical clustering. Left-hand labels indicate clusters that are commonly upregulated across *Rora* conditions (C-up) or specifically upregulated in Low/High/ΔNTD (Sp-up). MEFs and ESCs are shown as anchors. L, Rora^Low^ system. H, Rora^High^ system. ΔN, Rora^ΔNTD^ system. Right-hand labels show gene counts (*n*). Genes were considered significant if they met *p* < 0.05 with an absolute fold-change threshold of |log2(fold change)| > 1. (D) Heatmap of genes downregulated in Rora^Low^, Rora^High^, and Rora^ΔNTD^ relative to dsRed. Genes were grouped into 15 Down-clusters (DC1-DC15). Left-hand labels indicate clusters that are commonly downregulated across *Rora* conditions (C-D) or specifically downregulated (Sp-D). L, Rora^Low^ system; H, Rora^High^ system; ΔN, Rora^ΔNTD^ system. Genes were considered significant if they met *p* < 0.05 with an absolute fold-change threshold of |log2(fold change)| > 1. (E) GO (Biological Process) enrichment for DC1 genes from (D). Bars show −log10(*p* value) from Fisher’s exact test; numbers in parentheses denote gene counts. (F) GO (Biological Process) enrichment for DC13 genes from (D). Bars show −log10(*p* value) from Fisher’s exact test; numbers in parentheses denote gene counts. (G) Volcano plots of differentially enriched proteins at D3 (Rora^ΔNTD^ vs. Rora^High^). IP-MS experiments were performed in triplicates. A two-sided *t* test with Benjamini-Hochberg correction was applied; thresholds were *p*.adjust<0.05 and fold change ≥2. (H) Volcano plots of differentially enriched proteins at D3 (Rora^High^ vs. dsRed). A two-sided *t* test with Benjamini-Hochberg correction was applied; thresholds were *p*.adjust<0.05 and fold change ≥2. (I) Heatmap showing D3 IP-MS protein intensities for proteins that are differentially enriched in at least one pairwise comparison among dsRed, Rora^Low^, Rora^High^, and Rora^ΔNTD^ (biological triplicates per condition). Differential enrichment was assessed in DEP2 using Strimmer’s q value (*t*) for FDR control; proteins were considered significant with adjusted *p* < 0.05 and |log2 fold change| ≥ 1. Proteins were grouped into P1–P6 by unsupervised hierarchical clustering based on their abundance patterns across conditions.

To define *Rora*-dependent programs during OKS reprogramming, we performed bulk RNA sequencing (RNA-seq) at D0, D3, D5, and D7 under four conditions—dsRed control, Rora^Low^, Rora^High^, and Rora^ΔNTD^ with MEFs and embryonic stem cells (ESCs) as references (Figure 2A). We also performed immunoprecipitation-mass spectrometry (IP-MS) on D3 lysates from these four conditions (Figure 2B) to profile RORA-associated protein networks. Here, Rora^Low^ denotes the viral input yielding the peak OCT4-GFP^+^ colony number (≈100 μL in Figure 1E), Rora^High^ denotes a dose that significantly suppresses colony formation relative to 0 μL (≈250 μL in Figure 1E), and Rora^ΔNTD^ was dosed to match Rora^Low^. To enable quantitative cross-experiment comparison, we additionally report the corresponding viral genome input (copies per well) for each dose in Table S1. Consistent with these operational definitions, RNA-seq confirmed graded *Rora* transcript levels over the time course, with Rora^High^ exhibiting the highest expression (Figure S2G).

Using the dsRed trajectory as the reference, we identified genes differentially expressed in the *Rora* arms and organized them into 15 “Up-clusters” (UC1–UC15) and 15 “Down-clusters” (DC1–DC15) by hierarchical clustering (Figures 2C and 2D). The left margin shows the cluster class (C-up/C-down for changes common to all *Rora* arms; sp-Low/sp-High/sp-ΔNTD for arm-specific changes). The right margin shows the number of genes in each cluster (n).

Among the Up-clusters, UC1–UC3 were commonly elevated across Rora^Low^, Rora^High^, and Rora^ΔNTD^ relative to dsRed and were enriched for developmental and metabolic processes by Gene Ontology (GO) (Figures S3A–S3C). By contrast, UC13 represented a High-specific module with GO terms related to tissue and cell development (Figure S3D), consistent with a dosage-dependent activation of differentiation programs in Rora^High^.

Down-clusters captured contraction of somatic programs and selective immune repression. DC1–DC3 (common down across *Rora* conditions) were enriched for leukocyte migration, extracellular matrix organization/structure, and related motility terms (Figures 2E, S3E, and S3F), reflecting loss of fibroblast identity during reprogramming. In contrast, the High-specific DC13–DC15 modules were strongly enriched for negative regulation of viral processes, defense/response to virus, regulation of viral life cycle/genome replication, and negative regulation of type I IFN-mediated signaling (Figures 2F, S3G, and S3H).

Consistently, IFN-stimulated genes (ISGs)—including *Ifit1*/*Ifit3*, *Isg15*, *Oas1a*/*Oas1c*, *Oasl1*/*Oasl2*, *Ifi44*/*Ifi47*, *Gbp7*, *Dhx58*/*Ddx60*, *Ifih1*, *Irf7*, *Rsad2*, *Mx2*, and *Eif2ak2*—were reduced most strongly in Rora^High^, intermediate in Rora^Low^, and partially restored in Rora^ΔNTD^ across the time course. Gene-level statistics are provided in Table S2.

### Protein interaction profiling supports dosage-dependent immune modulation

To relate dosage to RORA-associated protein networks, we analyzed the D3 IP-MS datasets from dsRed, Rora^Low^, Rora^High^, and Rora^ΔNTD^ cultures.

To test whether the high-dose phenotype depends on the NTD, we compared Rora^ΔNTD^ with Rora^High^ using volcano plots (two-sided *t* test with Benjamini-Hochberg correction; p.adjust <0.05; fold change ≥2) (Figure 2G). A broad set of innate antiviral/IFN-responsive proteins shifted toward Rora^ΔNTD^ (positive log_2_fold change), indicating higher abundance in Rora^ΔNTD^ and relief of the high-dose repression. Representative examples include SLFN5, EPSTI1, IFI204, and SLC2A1. Proteins elevated in Rora^High^ (negative log_2_fold change; blue) were enriched for transcription/chromatin factors, consistent with a distinct high-dose state. Thus, the NTD is required for high-dose suppression of antiviral pathways, in agreement with the DC13-DC15 GO signatures and the partial restoration observed for Rora^ΔNTD^ across the time course.

We next defined proteins enriched in Rora^High^ relative to the negative control using volcano plots (two-sided *t* test with Benjamini-Hochberg correction; p.adjust <0.05; fold change ≥2) (Rora^High^ vs. dsRed; Figure 2H). As expected, RORA was among the most strongly enriched proteins in the Rora^High^ pull-down. Beyond RORA, Rora^High^-enriched candidates included multiple nuclear transcription/chromatin-associated factors, such as MECP2, PARP2, POLB, TFEB/TFE3, TFAM, and INO80/INO80C, as well as additional high-dose candidates including PBX3, RUNX1/RUNX2, TFAP2C, SATB2, and ACTR8. Together, these Rora^High^-biased interactors suggest that high-dose *Rora* engages a distinct nuclear regulatory environment and nominate candidates that could interface with the downstream pathway outputs described elsewhere in the study.

To assess dose dependence, we compared Rora^High^ with Rora^Low^ (Figure S3I). Immune/ISG-linked proteins (e.g., SLFN5, EPSTI1, and IFI204) were biased toward Rora^Low^, whereas Rora^High^ favored nuclear regulatory candidates (e.g., PBX3, DDX39A, MECP2, POLG2), consistent with dose-dependent remodeling of the RORA interactome.

To organize global interaction patterns across conditions, proteins significant in at least one pairwise comparison were grouped into six modules (P1–P6) by unsupervised clustering and visualized as a heatmap (Figure 2I; Table S3). This analysis resolves modules with distinct condition biases, including modules preferentially enriched in Rora^High^ and modules attenuated upon ΔNTD, consistent with NTD-sensitive interactome features.

Focusing on a Rora^High^-enriched subset (Figure S3J), we observed coordinated elevation of candidates linked to cell-state regulation and transcriptional control (ZEB1/2, PBX3, ZNF516, ZBTB45) as well as extracellular matrix components (COL4A1/COL4A2) in the Rora^High^ condition, with reduced abundance upon ΔNTD. This pattern is consistent with the idea that high-dose *Rora* engages interaction programs associated with non-productive state features during OKS reprogramming (including differentiation/epithelial-mesenchymal transition [EMT]-like and structural programs), aligning with the High-specific transcriptomic signatures and functional phenotypes described elsewhere.

Finally, GO enrichment analysis of the IP-MS modules (Figure S3K) highlighted two dominant themes. P2 + P3 + P4 were enriched for DNA replication, telomere maintenance, DNA damage response/repair, and chromatin remodeling, consistent with a nuclear genome-maintenance/chromatin regulatory program captured in the interactome. In contrast, P1 + P5 were enriched for terms linked to epithelial apoptosis regulation, EMT-associated processes, muscle differentiation, and nucleosome organization, suggesting separable modules that map to cell-state transition programs.

In sum, transcriptome and interactome analyses converge on a dosage-graded suppression of antiviral/IFN programs by *Rora*, most pronounced in Rora^High^ and partially relieved by deletion of NTD, alongside broad regression of somatic extracellular matrix/motility modules characteristic of fibroblast identity.

### Dose and domain dependence of RORA chromatin engagement and regulatory output

**Figure 3:**
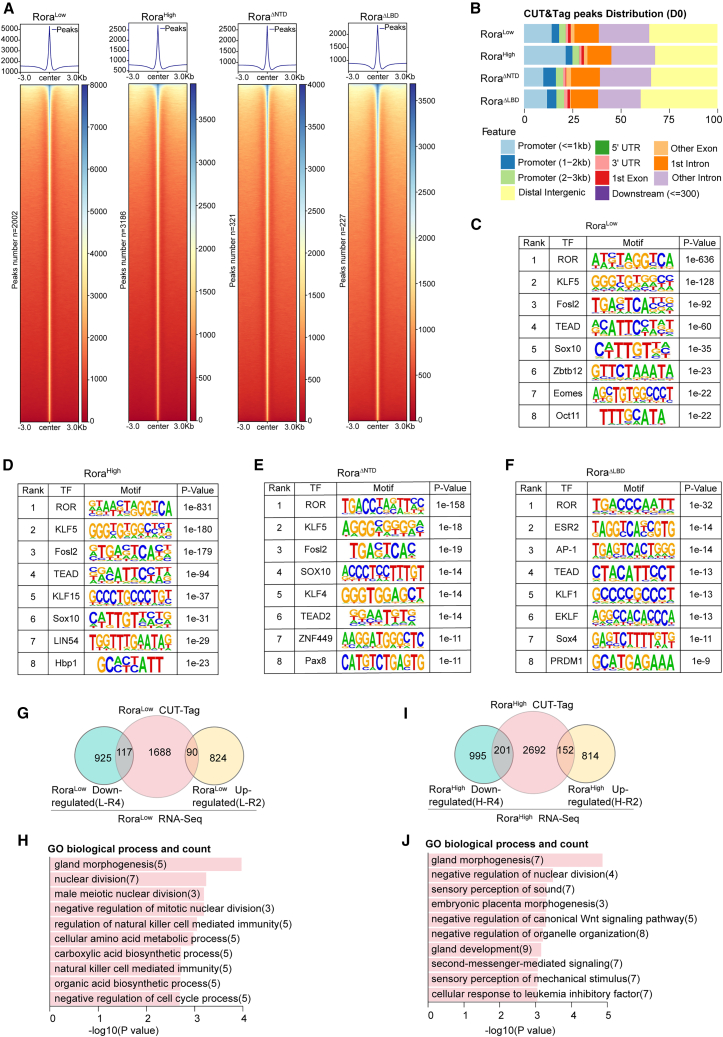
*Rora* dosage-dependent CUT&Tag landscapes and integrative functional enrichment during OKS-mediated reprogramming (A) Heatmap of CUT&Tag data at D3 from Rora^Low^, Rora^High^, Rora^ΔNTD^ (N-terminal domain deletion), or Rora^ΔLBD^ (ligand-binding domain deletion), respectively, showing all binding peaks centered on the peak region within a ±3-kb window around the peak. (B) Genome distribution of the location for Rora^Low^/Rora^High^/Rora^ΔNTD^/Rora^ΔLBD^-occupied peaks relative to the nearest annotated gene. (C–F) Transcription factor motif enrichment of Rora^Low^/Rora^High^/Rora^ΔNTD^/Rora^ΔLBD^-binding peaks. *De novo* and known motif analyses were performed using HOMER (hypergeometric test with Benjamini-Hochberg FDR correction), and the −log10(*p*-value) for the motif is shown. (G) Overlap of CUT&Tag peaks with RNA-seq modules in the Rora^Low^ system. (H) GO (Biological Process) enrichment for up-regulated overlapping genes in (G). Bars show −log10(*p* value) from Fisher’s exact test; numbers in parentheses denote gene counts. (I) The overlapping genes compared by CUT&Tag and RNA-seq data of Rora^High^ system. (J) GO (Biological Process) enrichment for up-regulated overlapping genes in (I). Bars show −log10(*p* value) from Fisher’s exact test; numbers in parentheses denote gene counts.

To define how RORA dosage and domains shape chromatin engagement during OKS reprogramming, we performed CUT&Tag with FLAG-tagged *Rora* constructs (Rora^Low^, Rora^High^, Rora^ΔNTD^, Rora^ΔLBD^) (Figure S4A), and ATAC-seq across the reprogramming time course (Figure S4B), and integrated the data with RNA-seq. Aggregate plots and heatmaps centered on peak summits showed sharp, focal RORA binding across thousands of loci. Peak numbers increased from Rora^Low^ to Rora^High^ (*n* = 2,002 and *n* = 3,186), indicating a dose-dependent expansion of the cistrome. Deleting the NTD (ΔNTD) or the LBD (ΔLBD) markedly reduced confident peaks (*n* = 321 and *n* = 227) and weakened centered signal intensity, demonstrating domain dependence of genome-wide occupancy (Figure 3A). These data showed that RORA occupancy scales with dose and requires intact NTD or LBD.

An initial analysis of genomic peak distribution revealed that, across conditions, peaks mapped to both promoter-proximal and non-coding regions, with large fractions in distal intergenic and intronic bins (Figure 3B). This suggests that RORA may regulate gene expression not only by binding to promoters but also by engaging distal regulatory elements, e.g., enhancers and potentially silencers, in distal intergenic regions and introns. Increasing dose (from Rora^Low^ to Rora^High^) did not appreciably alter the distribution across feature classes, whereas Rora^ΔNTD^ and Rora^ΔLBD^ showed a relative loss of promoter-proximal peaks and a gain in intronic/distal categories. Exact proportions for each feature bin are provided in Table S4, which quantitatively confirms the domain-dependent shift away from promoters.

*De novo* motif discovery recovered the ROR monomer motif (RORE) as the top motif in all datasets, with enrichment magnitude Rora^High^ > Rora^Low^ ≥ Rora^ΔNTD^ ≥ Rora^ΔLBD^. Rora^Low^ and Rora^High^ shared KLF5/AP-1 (FOSL2)/TEAD motifs, and High additionally showed KLF15/LIN54/HBP1, consistent with dose-dependent shifts in co-motif enrichment and binding-site selection rather than wholesale retargeting. Notably, KLF5 and FOSL2 (AP-1) were strongly enriched in Rora^Low^, Rora^High^, and Rora^ΔNTD^, but not in Rora^ΔLBD^ (Figures 3C–3F), suggesting that RORA’s pro-reprogramming activity may require LBD-dependent cooperation with KLF/AP-1 factors. Rora^ΔLBD^ displayed weakened RORE enrichment and increased non-RORE motifs (ESR2/AP-1/TEAD/KLF1), consistent with low-specificity, partner-driven binding, and its lack of pro-reprogramming activity. Rora^ΔNTD^ retained RORE recognition but shifted toward KLF4/KLF5/TEAD2, matching the loss of the high-dose inhibitory program seen transcriptionally (Figure 2G).

We intersected genes annotated by Rora^Low^ or Rora^High^ peaks with RNA-seq differential modules (L-R2/H-R2: upregulated set; L-R4/H-R4: downregulated set) defined for each condition. The Venn diagrams show the overlap sets, and GO on these overlaps includes developmental programs and negative regulation of canonical WNT signaling (Figures 3G–3J). Figures S4C and S4D provide the RNA-seq heatmaps for the R2/R4 module used in Figures 3G–3I, serving as a supplementary visualization of what R2/R4 comprises across conditions/time. Figure S4E shows the three-way Venn of CUT&Tag peaks (Rora^Low^, Rora^High^, and Rora^ΔNTD^) and GO of shared peaks, which highlight WNT-related categories, suggesting that WNT-linked loci are bound across conditions, while expression outputs depend on dosage and domain context.

To distinguish features shared by the pro-reprogramming conditions (Rora^Low^ and Rora^ΔNTD^) from those associated with the inhibitory Rora^High^ state, we examined Rora^High^-specific CUT&Tag binding events. GO analysis of peak-linked genes from Rora^High^-only sites revealed enrichment for signaling and cytoskeletal/organization-related processes (Figure S4E), consistent with a redistribution of RORA occupancy under high dosage. To link these high-dose binding events to transcriptional outputs, we intersected Rora^High^-only peak-linked genes with Rora^High^-regulated differentially expressed genes and analyzed the overlap (Figure S4F). The resulting candidate set was enriched for terms including regulation of canonical Wnt signaling (Figure S4G), supporting the interpretation that, while multiple *Rora* conditions occupy regulatory elements near Wnt-associated loci, the high-dose state shows a stronger association with Wnt-linked transcriptional outputs.

To compare RORA occupancy across conditions in an unbiased manner, we compiled a union peak set from D0 CUT&Tag across Rora^Low^, Rora^High^, Rora^ΔNTD^, and Rora^ΔLBD^ and performed unsupervised clustering based on log-transformed pileup signals (Figure S5A). We then linked peaks to nearby genes and integrated these peak-associated gene sets with RNA-seq differential expression to evaluate whether occupancy modules preferentially map to transcriptional activation (UP) or repression (DOWN).

Peak-associated genes from the dominant occupancy module (C3) showed substantial overlap with both Rora^High^ UP- and DOWN-regulated gene sets (Rora^High^ vs. dsRed; Figure S5B), consistent with a broad high-dose binding state that accompanies both transcriptional activation and repression. Notably, genes associated with C5 overlapped preferentially with Rora^High^ UP-regulated genes when comparing Rora^High^ vs. Rora^ΔNTD^ (Figures S5C and S5D), supporting that NTD-dependent high-dose occupancy features are more tightly coupled to transcriptional activation. In contrast, C6 showed only modest overlap with Rora^Low^ regulated genes (Rora^Low^ vs. dsRed; Figure S5E), consistent with Rora^Low^ primarily tuning existing reprogramming trajectories rather than driving a large, unique occupancy-defined gene program. GO analysis of the occupancy modules highlighted Wnt-related terms and cytoskeleton/adhesion-linked processes among the peak-associated gene sets (Figure S5F), linking RORA occupancy patterns to pathways emphasized in the main model.

To further connect the limited Rora^ΔNTD^ occupancy to transcriptional outputs, we integrated Rora^ΔNTD^ CUT&Tag peak-linked genes with Rora^ΔNTD^ regulated DEGs from the RNA-seq time course. This analysis revealed a restricted overlap between ΔNTD-associated binding events and ΔNTD-responsive transcripts (Figures S6A and S6B), consistent with the relatively small ΔNTD peak set. GO analysis of the overlapping candidates showed modest enrichments dominated by broad signaling-related terms, including small GTPase-mediated signal transduction, along with additional context-dependent categories (Figure S6B). Together, these results suggest that, despite reduced genome-wide occupancy, Rora^ΔNTD^ can couple a compact set of retained binding events to measurable transcriptional changes, consistent with its strong pro-reprogramming phenotype.

Together, CUT&Tag and motif analyses indicate that RORA engages RORE-anchored elements at promoters and distal regulatory regions; dose increases cistrome strength and shifts co-motif usage; an intact LBD is essential for pro-reprogramming, whereas the NTD is required for the high-dose inhibitory arm.

### RORA dosage and domains govern chromatin accessibility dynamics during OKS reprogramming

**Figure 4:**
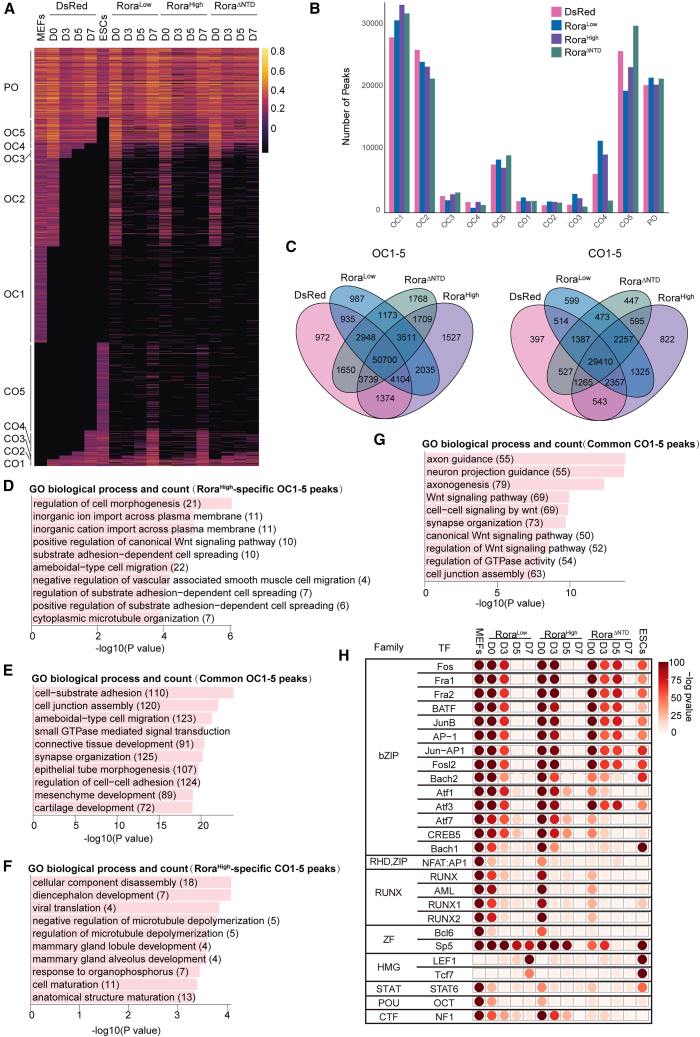
*Rora* dosage remodels chromatin accessibility dynamics and motif landscapes during OKS-mediated reprogramming (A) Heatmap of ATAC-seq signal across dynamic chromatin accessibility modules defined relative to the dsRed trajectory from MEFs (start) to ESCs (endpoint). ATAC peaks were called per sample (MACS2, q < 0.05) and merged across all samples to generate a union peak set. For each union interval, accessibility (open/closed) was assigned at each time point in the dsRed control, and intervals were classified into three classes based on MEF to ESC state transitions: PO (open in both MEFs and ESCs), OC (open in MEFs and closed in ESCs; “open-to-close”), and CO (closed in MEFs and open in ESCs; “close-to-open”). OC and CO intervals were further subdivided into timing submodules (1–5) based on the earliest time point at which the interval adopts and thereafter maintains the ESC-endpoint state (e.g., CO1 opens by D0 and remains open, CO2 opens by D3 and remains open, similarly for OC timing of closure). Rows are union intervals ordered by module; columns show MEFs, D0/D3/D5/D7, and ESCs for each condition. Color indicates normalized accessibility signal (scale shown). (B) Aggregate module membership counts for each condition. Using the same union peak set and the dsRed-defined module assignments in (A), we quantified, for each condition, the number of union intervals falling into each module (OC1–OC5, CO1–CO5, and PO) based on the condition’s time-course open/closed pattern toward the ESC endpoint. Thus, counts in (B) represent total numbers of intervals per module across the full time course, not peaks from a single time point. Because CO5 denotes late-opening intervals whose signal is expected to remain low until the endpoint, CO5 can appear weak across intermediate days in (A) while still contributing substantial module counts in (B). (C) Venn diagrams showing overlap of CO and OC peaks among dsRed, Rora^Low^, Rora^High^, and Rora^ΔNTD^ systems. (D) GO analysis of Rora^High^ specific OC1–OC5 peaks (*n* = 1,527). Bars show –log10(*p* value) from Fisher’s exact test; numbers in parentheses denote gene counts. (E) GO analysis of common OC1–OC5 peaks shared by Rora^Low^, Rora^High^, and Rora^ΔNTD^ systems after excluding dsRed (*n* = 3,511). Bars show −log10(*p* value) from Fisher’s exact test; numbers in parentheses denote gene counts. (F) GO analysis of Rora^High^-specific CO1–CO5 peaks (*n* = 822). Bars show –log10(*p* value) from Fisher’s exact test; numbers in parentheses denote gene counts. (G) GO analysis of common CO1–CO5 peaks shared by Rora^Low^, Rora^High^, and Rora^ΔNTD^ systems after excluding dsRed (*n* = 2,257). Bars show −log10(*p* value) from Fisher’s exact test; numbers in parentheses denote gene counts. (H) Motif enrichment analysis of Rora^Low^, Rora^High^, and Rora^ΔNTD^ peaks at D0, D3, D5, and D7 during iPSC induction. Peaks from dsRed at each corresponding time point were removed as background. *De novo* and known motif analyses were performed using HOMER (hypergeometric test with Benjamini-Hochberg FDR correction), and the −log10(*p* value) for motif is shown.

Chromatin remodeling is essential for cell-fate conversion (Chen et al., 2024; Wang et al., 2023). To chart chromatin accessibility dynamics under distinct RORA states, we performed ATAC-seq at D0, D3, D5, and D7 in dsRed controls and in cells expressing Rora^Low^, Rora^High^, or Rora^ΔNTD^, with ESCs as a reference. Peaks were classified by their behavior between MEFs and ESCs into three classes—PO (open in both), OC (open in MEFs then closed in ESCs), and CO (closed in MEFs then open in ESCs)—and OC and CO were further subdivided by timing into OC1–OC5 and CO1–CO5 to capture progressive closing and opening. Heatmaps revealed constitutively accessible PO regions and dynamic OC/CO modules across the time course (Figure 4A). Quantification of module membership across the full time course showed that Rora^Low^ increased representation of closing modules (OC; MEF-open loci that become closed toward ESC) and reduced the latest-opening CO5 module relative to dsRed, consistent with accelerated consolidation of ESC-endpoint chromatin states and timely silencing of somatic loci (Figure 4B). In contrast, Rora^High^ expanded OC1 but retained more peaks in CO5 than Rora^Low^, indicating imbalanced remodeling with incomplete closure of somatic chromatin. Rora^ΔNTD^ largely mirrored the high-dose pattern for OC1 and showed the least closure (highest peak counts) in CO5 together with a marked reduction in CO4, indicating that the NTD is required for proper consolidation of closing regions. PO peaks remained broadly similar across conditions (Figures 4A and 4B). Venn diagrams for opening (CO1–CO5) and closing (OC1–OC5) modules revealed a large common core of peaks across dsRed, Rora^Low^, Rora^High^, and Rora^ΔNTD^, together with condition-skewed subsets, indicating that RORA primarily modulates the extent and timing of accessibility rather than retargeting to entirely new loci. Moreover, Venn analysis of OC1–OC5 peaks (Figure 4C) identified 1,527 peaks unique to Rora^High^. GO annotation of these High-specific closing-module peaks highlighted terms such as regulation of cell morphogenesis and positive regulation of canonical Wnt signaling (Figure 4D). After excluding the dsRed control, 3,511 OC1–OC5 peaks were shared by Rora^Low^, Rora^High^, and Rora^ΔNTD^; GO analysis of this shared set was enriched for tissue development processes (Figure 4E). Taken together, these data indicate that, although CUT&Tag shows that Rora^Low^, Rora^High^, and Rora^ΔNTD^ all occupy regulatory regions near WNT genes and ATAC-seq identifies a common pool of accessible WNT-associated elements across conditions, the transcriptional output of WNT targets is preferentially suppressed in Rora^High^ (as supported by the RNA-seq/GO analyses), rather than by differences in accessibility alone.

For CO1–CO5 peaks unique to Rora^High^ (Figure 4C), GO terms concentrated in cellular component disassembly, microtubule (de)polymerization control, cell/anatomical structure maturation, and select organ development categories, together with viral translation/response to organophosphorus (Figure 4F). These annotations suggest that high-dose *Rora* engages additional structural turnover and stress response programs during chromatin opening.

By contrast, CO1–CO5 peaks shared by Rora^Low^, Rora^High^, and Rora^ΔNTD^ (Figure 4C) were enriched for axon/neurite guidance and axonogenesis, Wnt signaling pathway, canonical Wnt signaling pathway, regulation of Wnt signaling, and GTPase/junction assembly functions (Figure 4G).

Motif enrichment across time points uncovered two dose/domain-sensitive axes (Figure 4H). bZIP/AP-1 family motifs (FOS, FRA1/2, BATF, JUNB, AP-1, ATF1/3/7, CREB5, and BACH1/2) were transiently elevated at D3–D5 in all conditions, with the strongest signals in Rora^High^, and Rora^ΔNTD^, indicating an AP-1-dominated opening phase that is amplified by high dose or loss of the NTD. In contrast, motifs associated with the WNT/TCF module (LEF1, TCF7) and the WNT target Sp5 increased as cultures approached pluripotency and were most prominent in ESCs, marking maturation toward an ESC-like state. RUNX motifs appeared early and waned over time.

To relate RORA chromatin binding to dynamic accessibility states, we intersected RORA CUT&Tag peaks with ATAC-defined PO/OC/CO modules (defined in Figure 4A) at D0, D3, and D5 across Rora^Low^, Rora^High^, and Rora^ΔNTD^. Aligning ATAC modules with CUT&Tag signal showed that RORA binding is predominantly detected within accessible chromatin, with strong signal over PO regions and appreciable occupancy within dynamic OC/CO modules (Figure S6C). Quantifying peak distribution confirmed that the majority of RORA CUT&Tag peaks fall into PO, with smaller fractions mapping to OC and CO, across conditions and time points (Figure S6D). To control for differences in module size, we further computed an ATAC-class-normalized occupancy metric (overlapped CUT&Tag peaks divided by the number of ATAC regions in each class), which similarly supported preferential occupancy of PO and measurable engagement of dynamic OC/CO regions during the early-to-mid reprogramming window (Figure S6E). These data provide a direct intersection-based linkage between RORA occupancy and ATAC-defined chromatin remodeling dynamics.

In summary, these data indicate that chromatin accessibility during reprogramming is dose sensitive and that the NTD is required to properly consolidate closing modules.

### *Rora* dosage modulates IFN-γ-signaling and WNT output during OKS-mediated reprogramming

**Figure 5:**
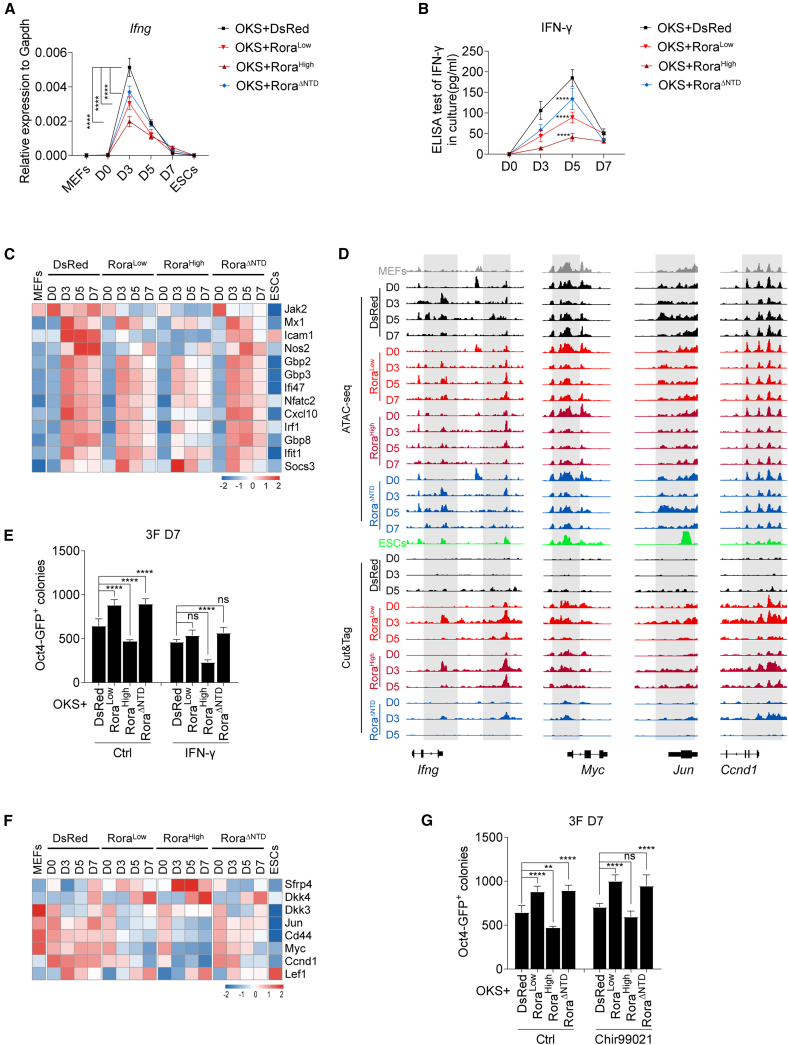
*Rora* dosage modulates the IFN-γ axis and WNT signaling during OKS-mediated reprogramming (A) RT-qPCR analysis of *Ifng* expression (normalized to *Gapdh*) across the indicated time points (D0, D3, D5, D7) in dsRed, Rora^Low^, Rora^High^, and Rora^ΔNTD^ conditions. Data are mean ± SD; two-way ANOVA, Tukey’s test; *n* = 3 biological replicates; significance as indicated. ^∗∗∗∗^*p* < 0.0001. (B) ELISA quantification of secreted IFN-γ in culture supernatants. Data are mean ± SD; two-way ANOVA, Tukey’s test; *n* = 3 biological replicates; significance as indicated. ^∗∗∗∗^*p* < 0.0001. (C) RNA-seq heatmap of selected IFN-γ-responsive genes across the OKS time course (MEFs, D0, D3, D5, D7, ESCs) in dsRed, Rora^Low^, Rora^High^, and Rora^ΔNTD^. (D) Genome browser views showing ATAC-seq accessibility and CUT&Tag signal at the *Ifng*, *Myc*, *Jun*, and *Ccnd1* loci for the conditions and time points indicated. (E) Effect of exogenous IFN-γ on OKS reprogramming efficiency (OCT4-GFP^+^ colony numbers at D7) in dsRed, Rora^Low^, Rora^High^, and Rora^ΔNTD^ backgrounds. Data are mean ± SD; one-way ANOVA, Holm-Šídák’s test; *n* = 3 independent experiments; significance as indicated. ^∗∗∗∗^*p* < 0.0001. ns, not significant. (F) RNA-seq heatmap of selected WNT pathway genes across the OKS time course and conditions as in (C). (G) Effect of WNT pathway activation with CHIR99021 on OKS reprogramming (OCT4-GFP^+^ colonies at D7) under dsRed, Rora^Low^, Rora^High^, and Rora^ΔNTD^ conditions. Data are mean ± SD; one-way ANOVA, Holm-Šídák’s test; *n* = 3 independent experiments; significance as indicated. ^∗∗^*p* < 0.01, ^∗∗∗∗^*p* < 0.0001, ns, not significant.

Prior analyses implicated immune pathways in the *Rora* response: RNA-seq GO terms (Figure 2F) and proteomics (Figure 2I) were enriched for IFN/antiviral programs, motif dynamics highlighted early AP-1 activity (Figure 4H), and RORA is reported to modulate immune responses (Hall et al., 2022; Wang et al., 2021). Moreover, innate immune signaling is known to influence somatic reprogramming. Because IFN-γ has been described as a barrier to reprogramming (Barrero et al., 2024; Guo et al., 2019), we directly measured *Ifng* mRNA and secreted IFN-γ in our system (Figures 5A and 5B). Quantitative reverse-transcription PCR (RT-qPCR) showed that *Ifng* transcripts peaked around D3 in dsRed controls and were significantly reduced in Rora^Low^, Rora^High^, and Rora^ΔNTD^ (Figure 5A). Consistently, ELISA of culture supernatants at D3 detected lower secreted IFN-γ in all three *Rora* conditions compared with dsRed (Figure 5B). A heatmap of IFN-γ-responsive genes from the RNA-seq time course revealed global attenuation of the pathway in *Rora* groups relative to dsRed (Figure 5C).

Genome browser tracks integrated ATAC-seq and CUT&Tag signals at the *Ifng* loci. CUT&Tag identified RORA-bound peaks near *Ifng* in Rora^Low^, Rora^High^, and Rora^ΔNTD^, whereas ATAC-seq showed reduced accessibility of these genes, consistent with transcript attenuation (Figure 5D).

Because IFN-γ is a known barrier to somatic reprogramming, we tested whether engaging this pathway would counteract *Rora*-mediated enhancement. Exogenous IFN-γ added during reprogramming reduced OCT4-GFP^+^ colony numbers and specifically abrogated the benefit conferred by Rora^Low^; colony counts in Rora^Low^ with IFN-γ approached dsRed levels. Rora^ΔNTD^ also lost its modest improvement in the presence of IFN-γ, whereas dsRed was minimally affected (Figure 5E). These results suggest that limiting IFN-γ signaling is part of the mechanism by which Rora^Low^ promotes reprogramming.

RORA has been linked to WNT pathway regulation in several contexts (Lee et al., 2010, 2021). In our data, set-based and motif analyses pointed to WNT-related categories (Figures 3J, 4E, and S4E). We, therefore, examined WNT components/targets across conditions and asked whether WNT output differs at high *Rora* dosage and whether activating WNT can modify the phenotype. RNA-seq heatmaps of WNT pathway genes showed that several key components and targets are selectively downregulated in Rora^High^ (Figure 5F). Consistently, ATAC-seq in Figure 5D revealed decreased accessibility near canonical WNT targets (*Myc*, *Jun*, and *Ccnd1*) in Rora^High^, despite detectable Rora^Low^, Rora^High^, and Rora^ΔNTD^ binding by CUT&Tag. To test causality, we activated WNT signaling with the GSK3 inhibitor CHIR99021. CHIR99021 significantly rescued the colony-forming defect of Rora^High^, restoring OCT4-GFP^+^ colonies toward dsRed levels, while having minimal effect on Rora^Low^ or Rora^ΔNTD^ (Figure 5G). Together, these data indicate that excess *Rora* blunts WNT pathway output, and WNT activation can partially rescue the high-dose inhibition. In the Rora^High^ condition, WNT pathway output is reduced (Figure 5F), and transcripts within innate antiviral/IFN modules are also diminished (Figures 2F and 2G). Prior work describes crosstalk between WNT and innate antiviral signaling (Dai et al., 2024; Hillesheim et al., 2014; Yang et al., 2010), although the directionality can be context specific. In our system, these pathways change concordantly with dose: high *Rora* coincides with lower WNT output and lower ISG expression, and CHIR99021-mediated WNT activation partially rescues reprogramming (Figure 5G). While this does not establish a direct causal chain from WNT to ISG repression, it suggests that dosage-dependent *Rora* effects converge on both pathways. Dissecting the mechanistic link will require targeted perturbations.

Collectively, the IFN-γ axis is broadly attenuated by *Rora* expression, and Rora^Low^ leverages this to enhance reprogramming. In contrast, Rora^High^ compromises reprogramming by suppressing WNT pathway output, a defect that can be functionally rescued by CHIR99021. These findings align with our chromatin analyses showing dose-dependent accessibility changes and domain-specific partner usage.

Together, these findings indicate that *Rora*’s impact on reprogramming is dose sensitive: DBD/LBD-dependent low dose enhances reprogramming in part by limiting IFN-γ signaling, whereas NTD-dependent high dose inhibits reprogramming with reduced WNT pathway output and weakening antiviral programs.

## Discussion

Our NR screen nominated the ROR subfamily as enhancers of OKS reprogramming and uncovered a biphasic *Rora* phenotype: Rora^Low^ increases, whereas Rora^High^ decreases, OCT4-GFP^+^ colony numbers. Domain analyses separated these arms: the DBD and LBD were required for the pro-reprogramming effect, while the NTD is required for high-dose inhibition (ΔNTD removes the inhibitory limb without abolishing enhancement). Conceptually, this places NR dosage and modular domains as orthogonal levers to tune reprogramming outputs, consistent with contemporary views of NR allostery/cofactor gating that couple ligand, dose, and domain context to chromatin regulation.

CUT&Tag indicates that RORA binds RORE-centered elements across conditions, arguing against wholesale retargeting. Instead, co-motif repertoires shift with dose and domain integrity: Rora^Low^ and Rora^High^ share KLF/AP-1/TEAD motifs; Rora^High^ additionally features KLF15/LIN54/HBP1 signatures; and ΔLBD retains RORE yet fails to enhance colonies, consistent with loss of LBD-dependent co-activator engagement. These patterns align with recent work positioning AP-1 as a temporal gatekeeper of cell-identity transitions (including reprogramming) (Markov et al., 2021) and TEAD/YAP as modulators of pluripotency/cytoskeletal programs (Kroeger et al., 2025; Pagliari et al., 2021); LIN54/HBP1 are components/associates of repressive complexes (DREAM; WNT restraint) that constrain transcriptional outputs (Wang et al., 2022). Together, the data support a model in which dose shifts binding-site selection on a relatively fixed cistrome. ATAC-seq resolves opening (CO) and closing (OC) modules along the trajectory. Rora^Low^ accelerates opening of developmental/WNT circuitry and timely closure of somatic adhesion modules, whereas Rora^High^ and Rora^ΔNTD^ maintain AP-1-linked accessibility and incompletely silence somatic chromatin-coherent with AP-1’s early barrier/rewiring roles and TEAD/YAP’s impact on cytoskeletal/fate transitions (Kroeger et al., 2025; Markov et al., 2021; Pagliari et al., 2021).

Functionally, *Ifng*/IFN-γ—a recognized barrier in several reprogramming contexts—was reduced at the transcript and protein levels in *Rora* arms, consistent with improved colony output at Rora^Low^; RORA’s broader immunomodulatory roles (e.g., RORA dampening PD-L1 to enhance tumor immunosurveillance) underscore plausibility for immune-axis tuning in a context-dependent manner (Liu et al., 2024; Mok et al., 2024; Pick et al., 2024). However, the relationship between IFN/ISG programs and reprogramming output is unlikely to be strictly monotonic. Prior work supports context and stage-dependent effects of IFN-γ and innate immune signaling during reprogramming: IFN-γ can facilitate aspects of pluripotency acquisition in some settings, yet single-cell trajectory analyses suggest that IFN-γ-associated programs can also hinder late transitions to fully competent pluripotency, and innate immune signaling can contribute to chromatin remodeling (Barrero et al., 2024; Guo et al., 2019; Lee et al., 2012). Together, these findings are consistent with a model in which Rora^Low^/Rora^ΔNTD^ establishes a permissive immune balance compatible with productive trajectories, whereas Rora^High^ represents a qualitatively distinct high-dose state in which the strongest IFN/ISG repression coincides with additional high-dose-engaged programs (including Wnt-linked outputs highlighted by Figures S4F–S4G) that constrain colony formation.

Conversely, WNT pathway output was lower in Rora^High^, and the high-dose defect was rescued by CHIR99021, consistent with syntheses emphasizing stage- and dose-dependent WNT effects during fate transitions (Cevallos et al., 2018; Kimura et al., 2016; Li et al., 2023; Xue et al., 2025). In this framework, the partial rescue by CHIR99021 is consistent with the phase-dependent nature of Wnt/β-catenin signaling: Wnt activation can promote colony formation or early transitions but may not fully normalize downstream progression when other high-dose programs are engaged. Notably, WNT-annotated elements can remain accessible yet poised/repressed in Rora^High^, emphasizing that accessibility is necessary but not sufficient for activation-cofactor composition and repressive complexes likely determine transcriptional output.

In summary, we propose that *Rora* dosage shifts the composition/stoichiometry of RORA-centered complexes on shared ROREs. At low dose, LBD-dependent co-activator usage (with KLF/AP-1/TEAD partners) facilitates chromatin transitions and reduces IFN-γ tone, yielding more OCT4-GFP^+^ colonies. At high dose, an NTD-dependent complex (partners nominated by motifs/IP clusters, e.g., LIN54/HBP1) dampens WNT output and sustains AP-1-linked accessibility, yielding fewer colonies—a phenotype reversed by CHIR99021. This model is most parsimonious with stable RORE anchoring, dose/domain-specific motif repertoires, module-selective accessibility changes (not global squelching), and WNT-specific rescue.

### Limitations of the study

This study has some limitations. The hypothesis that dose-dependent *Rora* effects are parsimoniously explained by the recruitment of distinct co-regulators is supported by complementary genomic and proteomic evidence; however, direct validation of the critical interacting proteins is lacking. Establishing causality necessitates future studies employing targeted perturbations of the identified candidate partners. Furthermore, the functional analysis of *Rora* is confined to the mouse OKS reprogramming system. Consequently, the extent to which the dose-dependent phenomena and the underlying mechanisms can be extrapolated to different reprogramming factor combinations (e.g., OKSM) or across species (e.g., in human cells) is unclear. Key variables such as factor stoichiometry, species-specific co-factor expression, and contextual signaling pathways (like YAP/TEAD or IFN-γ) may critically influence the outcomes, highlighting the system-specific nature of our current findings.

Although our data support a model in which RORA dosage differentially modulates IFN-γ and Wnt signaling to shape reprogramming efficiency, the mechanisms underlying RORA-dependent IFN regulation remain unresolved. It is unclear whether RORA directly controls transcription of IFN pathway components or modulates IFN signaling indirectly through upstream or intersecting networks. Future studies employing pathway-focused binding enrichment analyses, comparative transcriptomic profiling under IFN or Wnt-modulating conditions, and loss-of-function experiments targeting specific IFN signaling intermediates will help clarify the hierarchy and directness of RORA’s interaction with these pathways.

## Methods

### Preparation of MEFs and cell culture

NCG and ICR mice used in this study were purchased from GemPharmatech, while OCT4-GFP (OG2) transgenic mice were obtained from the Jackson Laboratory. All mice were housed under specific pathogen-free conditions with a 12-h light/dark cycle and provided with adequate food and water. All animal experiments were conducted in accordance with the animal care guidelines of the Guangzhou Institutes of Biomedicine and Health (GIBH) and approved by the GIBH Animal Experiment Ethics Committee.

This passage describes the isolation and culture of MEFs. Embryos were obtained by mating 129S4/SvJaeJ female mice with OG2 transgenic males and harvested at D13.5 of gestation. After dissecting away the head, internal organs, and limbs, the remaining tissue was digested with trypsin to create a single-cell suspension, which was then plated on gelatin-coated dishes. Both the resulting MEFs and the Plat-E cells used in the study were cultured in DMEM medium supplemented with 10% fetal bovine serum (FBS).

iPSCs were cultured in iCD1 medium (Chen et al., 2011), a defined culture system containing key components such as 50 μg/mL vitamin C, 10 ng/mL basic fibroblast growth factor (bFGF), and the small molecule 3 μM CHIR99021. The full formulation comprised DMEM, 1%NEAA, 1%GlutaMax, 1% sodium pyruvate, 0.1 mM β-Me, 1% N2, 1% B27, 50 μg/mL vitamin C, TV, LiCl, 3 μM CHIR99021, 10 ng/mL bFGF, and mLIF.

### Plasmid construction and retrovirus production

The full-length coding sequences of mouse NR genes and other relevant factors were amplified from cDNA derived from embryonic stem cells or embryonic fibroblasts, and the amplified products were cloned into the pMXs expression vector (CloneExpress II One Step Cloning Kit). Retroviruses were produced using the polyethylenimine (PEI) method in Plat-E cells. Specifically, 7.5 × 10^6^ Plat-E cells were seeded in 10-cm dishes and cultured in medium supplemented with 10% FBS for 12–18 h prior to transfection. For transfection, 20 μg of plasmid was mixed with 1 mL of Opti-MEM I by inverting the tube 15 times, followed by incubation at room temperature for 5 min. Then, 60 μL of PEI reagent was added, and the mixture was inverted another 15 times and incubated at room temperature for 8 min. The resulting complex was combined with 9 mL of DMEM and added to the Plat-E cells. After 10 h, the medium was replaced with fresh DMEM containing 10% FBS. Retroviral supernatant was collected at 48 and 72 h post-transfection for subsequent cell infection.

### Gene overexpression experiments

All overexpression experiments in this study were performed using a retroviral transduction approach, with both the target cells and retroviruses prepared as described in the preceding sections. Prior to infection, 250 μL of medium containing 10% FBS was added to each well of a 24-well plate, followed by an equal volume of each retroviral supernatant. The mixture was then supplemented with 8 μg/mL polybrene to enhance transduction efficiency, and the cells were incubated for 24 h. Following two rounds of infection, the medium was replaced with iCD1 medium to complete the procedure.

### iPSC induction Protocol

MEFs were thawed and seeded at a density of 1 × 10^4^ cells per well in a 24-well plate 12–24 h prior to viral infection. At 48 h post-transfection, the viral supernatant was collected using a 10-mL syringe and filtered through a 0.45-μm membrane into a 15-mL centrifuge tube and the Plat-E cells were replenished with fresh culture medium containing 10% FBS. Subsequently, each well of the 24-well plate received 250 μL of medium supplemented with 10% FBS, an equal volume of each retroviral supernatant, and 8 μg/mL polybrene to enhance infection of MEFs. After two rounds of infection, the medium was replaced with iCD1 induction medium, designated as D0. GFP-positive colonies were counted at specified time points to assess reprogramming efficiency.

### RNA-seq and gene expression analysis

RNA sequencing was performed using the mm10 mouse reference genome with vM23 annotations. Raw sequencing reads were processed through the following workflow. Adapter trimming and quality control were performed using fastp (v.0.23.2) (Chen et al., 2018). Cleaned reads were aligned to the genome using Hisat2 (v.2.2.1) (Kim et al., 2019), and gene-level quantification was obtained via featureCounts (v.2.0.3) (Liao et al., 2014). Differential expression analysis was conducted with DESeq2 (v.1.38.0) (Love et al., 2014) using RPKM-normalized counts, and functional enrichment was assessed through GO using clusterProfiler (v.4.6.2) (Yu et al., 2012).

For the Rora^High^, Rora^Low^, and Rora^ΔNTD^ reprogramming systems, differential gene expression analysis was carried out using DESeq2. Initially, differential gene expression was analyzed relative to the dsRed control. Genes with inconsistent regulation patterns (e.g., opposing trends at different time points) within the same system were excluded, and the remaining genes were integrated to identify differentially expressed genes within each system. Subsequently, these genes were categorized into distinct clusters based on their relative expression levels (fold change = 2) in ESCs and MEFs. Finally, genes were ranked according to their highest expression levels (or lowest in the case of downregulated genes) at specific time points.

Comparative analyses between systems employed the following categories: L/H/dN C-up: genes upregulated in one system (e.g., Rora^Low^) without differential expression or downregulation in others; L/H C-up or L/dN C-up: co-upregulated genes in two systems; low Sp-up/High Sp-up: genes exclusively upregulated in Rora^Low^ or Rora^High^ systems. Analogous classifications (“C-D,” “Sp-D”) were applied to downregulated genes.

### CUT&Tag analysis

CUT&Tag data were processed using the nf-core/cutandrun pipeline (v.3.2.1) (Ewels et al., 2020) within the Nextflow framework (Di Tommaso et al., 2017).

Raw sequencing reads were aligned to the mm10 mouse genome assembly using Bowtie2. PCR duplicates were removed using Picard MarkDuplicates. Peaks were identified using MACS2 with a default false discovery rate (FDR) threshold of q < 0.05. Normalized genome coverage tracks in BigWig format were generated using deepTools bamCoverage with RPKM normalization. Motif analysis was performed using HOMER2 with default settings. All aforementioned steps were executed as default components of the pipeline.

All control samples (dsRed and IgG) exhibited no statistically significant peak enrichment following MACS2 8 analysis (Welch’s *t* test, *p* > 0.05), confirming minimal non-specific background signals. Consequently, experimental samples were analyzed without comparative normalization to controls. GO analysis was performed using CHIPseeker with a focus on regions within 3 kb of the transcription start site (TSS).

### ATAC-seq and chromatin accessibility analysis

The ATAC-seq data were processed using the nf-core/ATAC-seq pipeline (v.2.1.2) (Ewels et al., 2020) implemented via Nextflow (Di Tommaso et al., 2017).

Key steps included the following: sequencing reads were aligned to the mm10 mouse reference genome using Bowtie2 with the very-sensitive preset, mitochondrial reads (chrM) were excluded during alignment via the pipeline’s built-in filtering step, PCR duplicates were removed using Picard MarkDuplicates, open chromatin regions were identified using MACS2 with a default FDR threshold of q < 0.05, and normalized genome coverage tracks in BigWig format were generated using deepTools bamCoverage with RPKM normalization to account for differences in sequencing depth. All aforementioned steps were executed as default components of the pipeline.

Subsequently, motif enrichment was analyzed with HOMER2 findMotifsGenome.pl, and peak annotation was performed with the ChIPseeker (v.1.42.1) (Yu et al., 2015) R package focusing on regions ±3 kb from the TSS.

For dynamic chromatin accessibility analysis, peaks were called using MACS2 (Zhang et al., 2008). Peaks meeting the threshold (q < 0.05) were defined as “open,” while the remainder peaks were classified as “closed.” An integrated background file containing all peaks across samples was generated using BEDTools. Each sample’s peaks were then compared against this background to identify regions exhibiting transitions between chromatin states during reprogramming-from MEFs (starting point) to ESCs (endpoint). These transitions included “close-to-open,” “open-to-close,” or “permanently open” states. Regions undergoing such changes were considered indicative of chromatin remodeling associated with pluripotency acquisition; CO (close-to-open): regions closed in MEFs that became open during reprogramming, CO1: a subset of CO regions that remained open from D0 onward, OC (open-to-close): regions open in MEFs that became closed during reprogramming, PO (permanently open): regions consistently open across all time points.

In the analysis of chromatin accessibility and motif enrichment across the Rora^Low^, Rora^High^, and Rora^ΔNTD^ systems at different time points (D0, D3, D5, and D7), peak calling was performed using MACS2, with dsRed samples from corresponding time points serving as controls to assess the specific impact of individual transcription factors on chromatin accessibility. Motif enrichment analysis of the identified peaks was subsequently performed using HOMER2. Module membership counts shown in Figure 4B reflect aggregate numbers of union intervals assigned per module across the full time course, rather than peaks from a single time point. RORA motif enrichment was assessed and found to be significant.

### Mass spectrometry analysis

Peptide separation was performed on a nanoEase M/Z Peptide BEH C18 column (Waters, 186008795) over a 60-min gradient, followed by two wash steps, using an Easy-nLC 1200 system coupled online to a Fusion Lumos mass spectrometer (Thermo Fisher Scientific). Mass spectrometry data were acquired in a data-independent acquisition (DIA) mode with a 90-s dynamic exclusion window. The raw files were processed with DIA-NN software, searching against the Mouse Fasta database. Label-free quantification and match-between-runs algorithms were applied. Subsequent analysis and visualization of the protein groups output were conducted using the DEP2 R package, as previously described (Feng et al., 2023; Zhang et al., 2018).

### Statistical analysis

Data are reported as mean ± SD and were analyzed in GraphPad Prism 9. For single-factor multi-group comparisons, we used one-way ANOVA followed by either Dunnett’s test (multiple comparisons vs. control) or Holm-Šídák multiple comparisons (pre-specified pairwise contrasts). For factorial designs, we used two-way ANOVA; post hoc Tukey’s tests were applied for multiple comparisons. All tests were two-tailed with statistical significance set at *p* < 0.05; adjusted *p* values are reported where applicable. The exact test used, *p* values, n, and numbers of independent experiments are provided in the figure legends. No statistical methods were used to predetermine sample size. Data for RNA-seq were performed twice, data for ATAC-seq and Cut&Tag were performed once, and data for IP-MS were performed three times in this study.

## Resource availability

### Lead contact

Further information and requests for resources and reagents should be directed to Dr. Jing Liu (liu_jing@gibh.ac.cn).

### Materials availability

This study did not generate new unique reagents.

### Data and code availability

All data are freely available. The bulk RNA-seq, CUT&Tag, and ATAC-seq data have been deposited in the Genome Sequence Archive (GSA) at the National Genomics Data Center, China National Center for Bioinformation/Beijing Institute of Genomics, Chinese Academy of Sciences, under Bioproject accession number PRJCA046784. The dataset accession number is CRA030540 and is publicly accessible at https://ngdc.cncb.ac.cn/gsa. The mass spectrometric data have been deposited to the ProteomeXchange Consortium via the PRIDE partner repository with the dataset identifier PXD068806. Original code used in this study is available from the corresponding author upon reasonable request. Any additional information required to reanalyze the data reported in this paper is available from the lead contact upon request.

## Acknowledgments

We thank the lab members in GIBH for their kind help. We thank the CRMH and members in CRMH for their kind help. We thank the members of the Analytical Instrumentation Core in GIBH for their help in data collection. We thank Dr. Xiaofei Zhang and Dr. Ruona Shi for their assistance in IP-MS data collection and analysis. This work was supported by 10.13039/501100012166National Key R&D Program of China (2024YFA1107902); 10.13039/100014718National Natural Science Foundation of China (32370791 and 32200695); Science and Technology Projects in Guangzhou (2023A04J0725 and 2025A04J7111); Health@InnoHK Program launched by Innovation Technology Commission of the Hong Kong SAR, P. R. China; Guangdong Basic and Applied Basic Research Foundation (2022A1515012267); 10.13039/501100012245Science and Technology Planning Project of Guangdong Province (2023B1212060050 and 2023B1212120009); and 10.13039/501100002858China Postdoctoral Science Foundation (2025M772848).

## Author contributions

J.L. and H.W. initiated the project and designed the experiments. H.W., Yusha Li, and Y.W. performed the reprogramming experiments. H.W. conducted and performed the CUT&Tag, ATAC-seq, RNA-seq, and IP-MS experiments with Yusha Li. C.Y., Z.Z. and Y.Z. analyzed the data. Yusha Li, L.Z., S.L., and A.X. constructed the plasmids. R. Luo isolated the OG2-MEFs cell lines. H.W., Yusha Li, and C.Y. wrote the manuscript with critical suggestions from Yi Li, C.L, R. Lin., X.Z., J.G., J.W., S.Y., B.C., L.X., and M.K. J.L. supervised the whole study, conceived the whole study, wrote the manuscript, and approved the final version.

## Declaration of interests

The authors declare no competing interests.

## Supplemental information

Document S1. Figures S1–S6 and Table S5

Table S1. Viral titers and copies per well

Table S2. ISG expression (TPM) across the time course

Table S3. IP-MS Heatmap list

Table S4. Genomic distribution of CUT&Tag peaks

Document S2. Article plus supplemental information
