# Supplementary material for: Dose-resolved control of somatic reprogramming by Rora
Source: Stem Cell Reports. 2026 Apr 2;21(4):102870. doi: 10.1016/j.stemcr.2026.102870 (PMC13083791; doi:10.1016/j.stemcr.2026.102870)
Supplement: Document S1. Figures S1–S6 and Table S5 [file mmc1.pdf]

Stem Cell Reports, Volume *21*

Supplemental Information

Dose-resolved control of somatic reprogramming by *Rora*

Haiyun Wang, Yusha Li, Chunkou Yin, Zhen Zhang, Yixuan Wang, Yi Li, Chuang Li, Runxia Lin, Xiaoli Zhang, Jing Guo, Rongping Luo, Shumin Li, Lv Zhang, Yingting Zhuang, Anchun Xu, Jiani Wan, Lizhan Xiao, Bailing Chen, Shengyong Yu, Manish Kumar, and Jing Liu

Supplemental Information

Figures S1–S6 (see below).

Figure S1

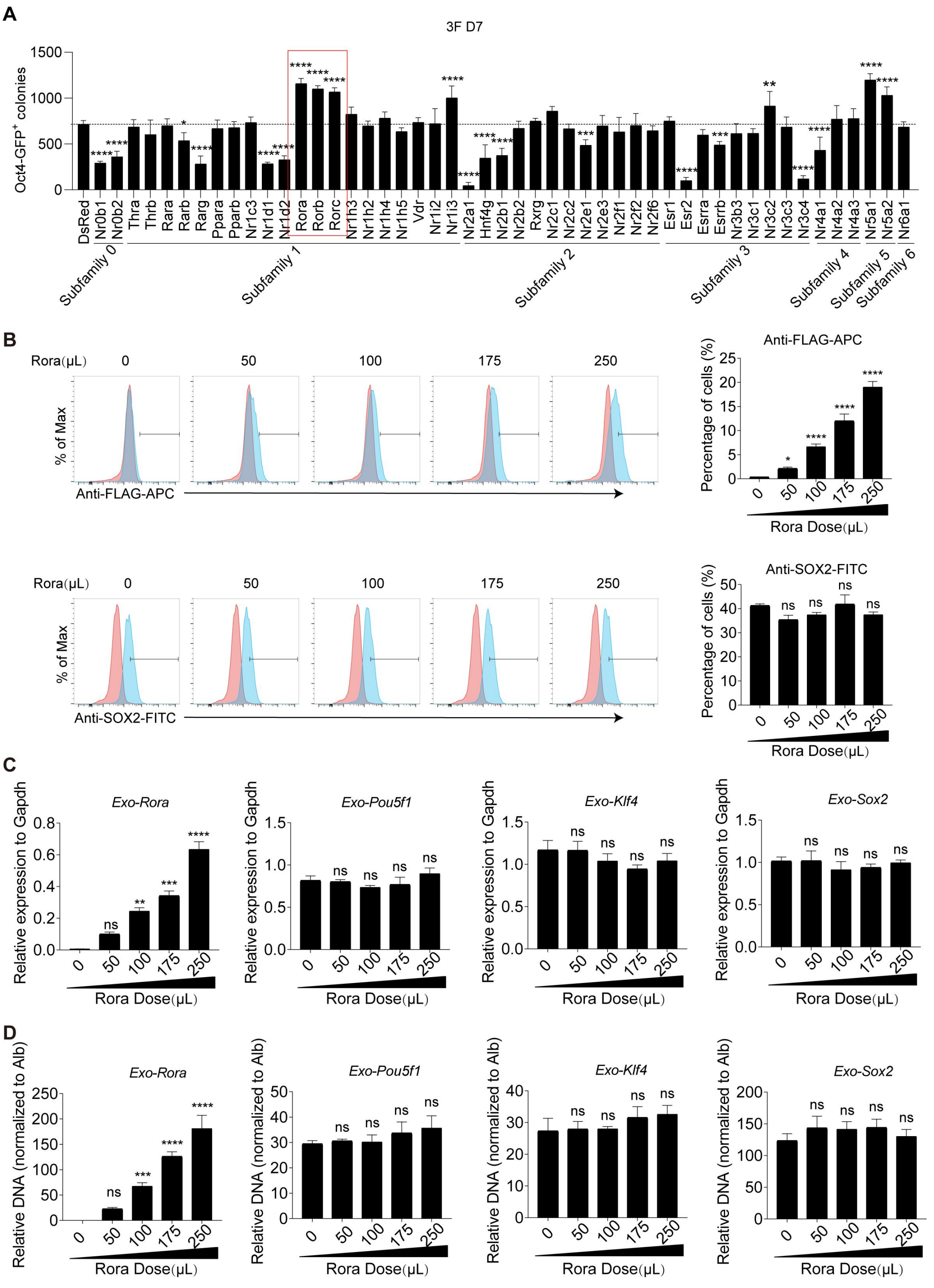

Figure S1. Primary nuclear receptor screen identifies *Rora* and validates dose-controlled *Rora* expression without altering OKS delivery. Related to Figure 1.

(A) Primary screen of 49 nuclear receptor (NR) family members in the OKS reprogramming system using mouse embryonic fibroblasts (MEFs). OCT4-GFP$^+$ colony numbers were quantified on Day 7 for each NR and compared with the DsRed control. Data are mean ± SD; one-way ANOVA, Dunnett's test; n=3 independent experiments; *P<0.05, **P<0.01, ***P<0.001, ****P<0.0001.

(B) MEFs were co-infected with OKS plus either DsRed control or *Rora*-Flag at the indicated *Rora* viral volumes. Measurements were performed 72 h after the second infection (D2). Flow cytometry showing intracellular FLAG (RORA-FLAG; anti-FLAG-APC) and SOX2 (anti-SOX2–FITC) signals across the *Rora* volume series. Representative histogram overlays are shown (left) and the percentage of positive cells is quantified (right). Data are mean ± SD; one-way ANOVA, Dunnett's test; n = 3 independent experiments; *P<0.05, ****P<0.0001. ns, not significant.

(C) RT-qPCR quantification of exogenous transcripts (Exo-*Rora*, Exo-*Pou5f1*, Exo-*Klf4*, Exo-*Sox2*) at D2, normalized to Gapdh, across the same *Rora* volume series. Data are mean ± SD; one-way ANOVA, Dunnett's test; n = 3 independent experiments; *P<0.05, ***P<0.001, ****P<0.0001, ns, not significant.

(D) Genomic qPCR quantification of vector DNA (VCN; Exo-*Rora*, Exo-*Pou5f1*, Exo-*Klf4*, Exo-*Sox2*) at D2, normalized to *Alb*, across the same *Rora* volume series. Data are mean ± SD; one-way ANOVA, Dunnett's test; n = 3 independent experiments; ***P<0.001, ****P<0.0001, ns, not significant.

Figure S2

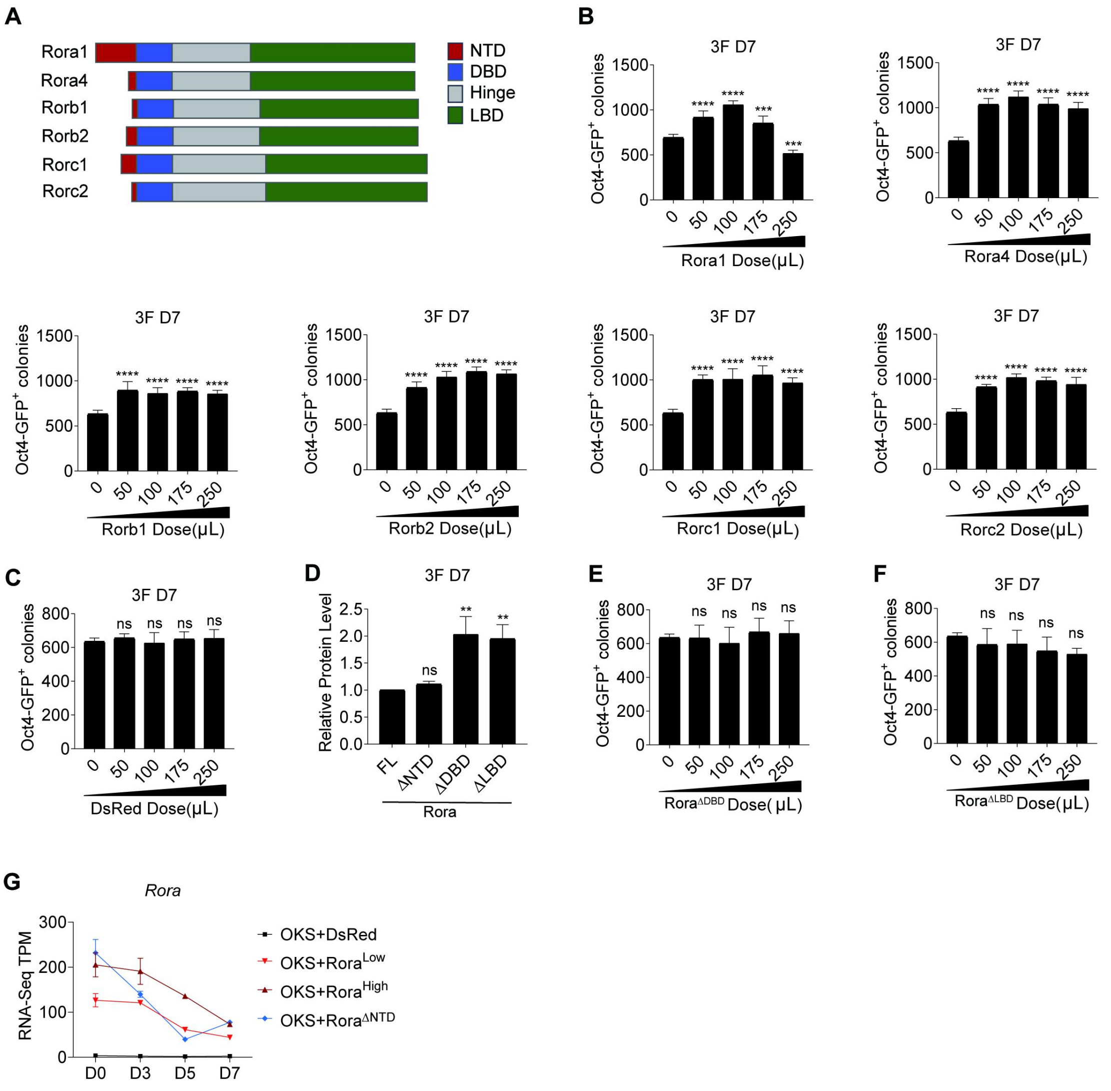

Figure S2. Dose-response profiles of the ROR subfamily and *Rora* domain-deletion series in OKS reprogramming. Related to Figure 1.

(A) Domain architecture of ROR subfamily proteins (*Rora*, *Rorb*, *Rorc*), highlighting the N-terminal domain (NTD), DNA-binding domain (DBD), hinge region, and ligand-binding domain (LBD).

(B) Dose-response analyses for ROR subfamily constructs (*Rora1*, *Rora4*, *Rorb1*, *Rorb2*, *Rorc1*, *Rorc2*). Data are mean ± SD; one-way ANOVA, Dunnett's test; n = 3 independent experiments; ***P<0.001, ****P<0.0001.

(C) Dose-response analyses for DsRed. Data are mean ± SD; one-way ANOVA, Dunnett's test; n = 3 independent experiments; ns, not significant.

(D) Densitometric quantification of (Figure 1K). Data are mean ± SD; one-way ANOVA, Dunnett's test; n = 3 independent experiments; ns, not significant, **P<0.01.

(E) Dose-response analyses for Rora$^{\Delta DBD}$. Data are mean ± SD; one-way ANOVA, Dunnett's test; n = 3 independent experiments; ns, not significant.

(F) Dose-response analyses for Rora$^{\Delta LBD}$. Data are mean ± SD; one-way ANOVA, Dunnett's test; n = 3 independent experiments; ns, not significant.

(G) RNA-seq TPM trajectories of *Rora* across the reprogramming time course (D0, D3, D5, D7) in OKS+DsRed, OKS+Rora^{Low}, OKS+Rora^{High}, and OKS+Rora$^{\Delta NTD}$ conditions.

Figure S3

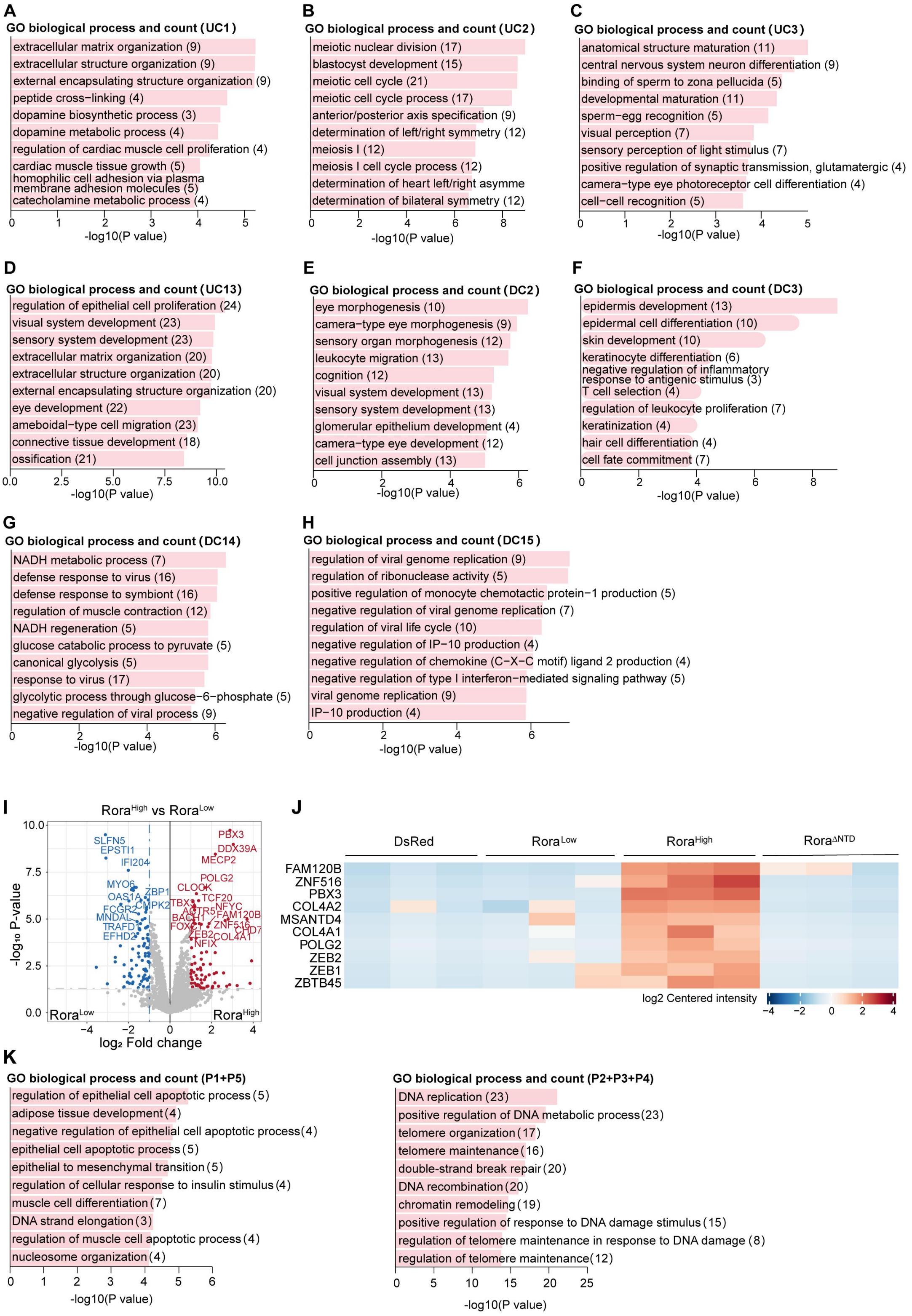

Figure S3. Dose-resolved transcriptomic and IP-MS pathway signatures associated with RORA states. Related to Figure 2.

(A-H) Gene Ontology (GO; Biological Process) enrichment for representative RNA-seq gene clusters from Figure 2C-D. Bars indicate -log10(P value) from Fisher’s exact test; numbers in

parentheses denote gene counts. UC, up-cluster; DC, down-cluster. (A) UC1; (B) UC2; (C) UC3; (D) UC13; (E) DC2; (F) DC3; (G) DC14; (H) DC15.

(I) Volcano plots of differentially enriched proteins at Day 3 ($Rora^{High}$ versus $Rora^{Low}$). A two-sided t-test with Benjamini-Hochberg correction was applied; thresholds were *p*.adjust<0.05 and fold-change ≥ 2.

(J) Heatmap of a $Rora^{High}$-enriched subset of proteins measured by Day 3 anti-FLAG IP-MS.

(K) GO enrichment for proteins in IP-MS clusters derived from Figure 2I. Bars show -log10(*P* value) from Fisher's exact test.

Figure S4

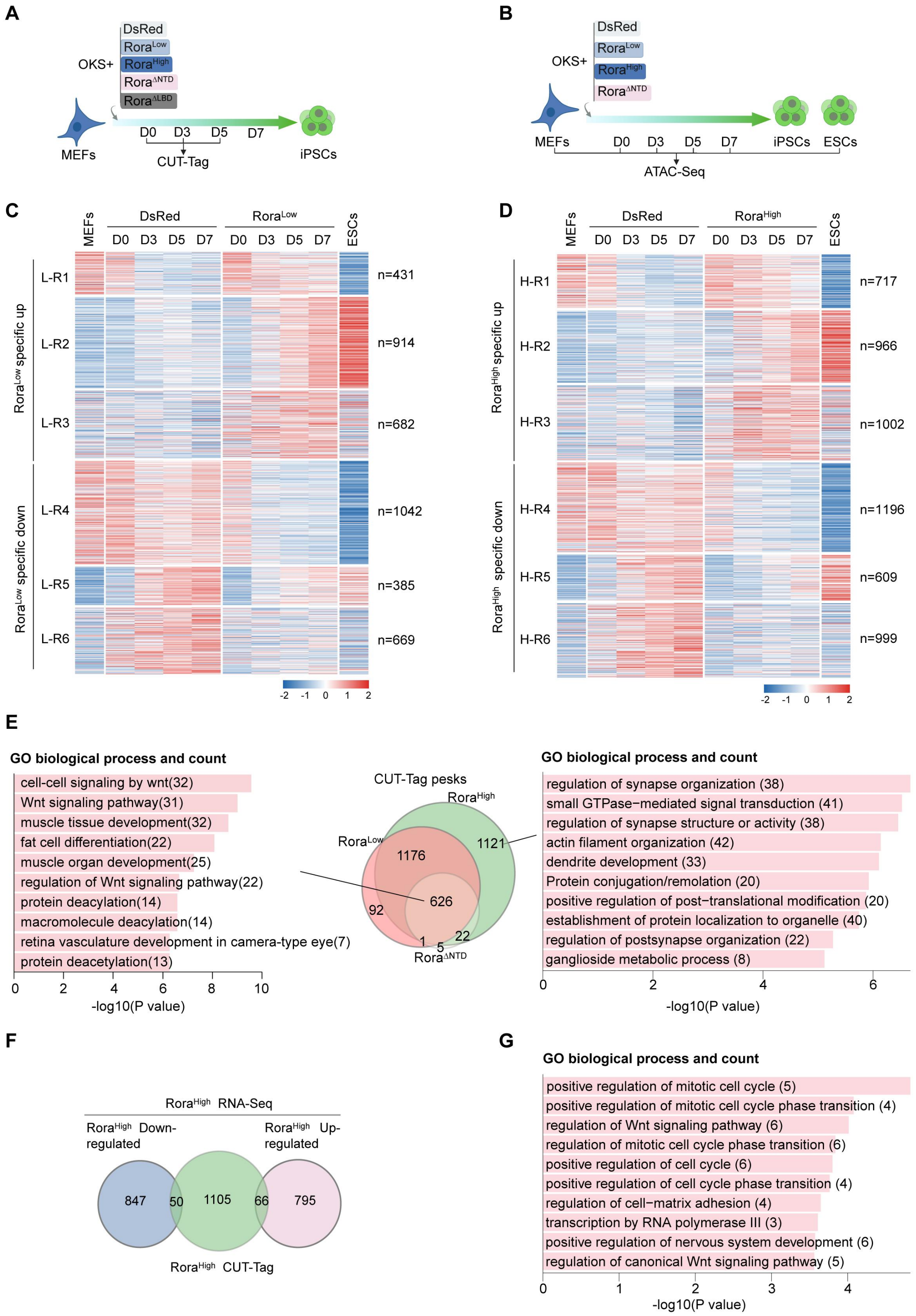

Figure S4. Extended analyses linking RORA occupancy to transcription. Related to Figure 3.

(A) CUT&Tag experimental design. Mouse embryonic fibroblasts (MEFs) were transduced with OKS together with DsRed (control), $Rora^{Low}$, $Rora^{High}$, $Rora^{\Delta NTD}$, or $Rora^{\Delta LBD}$. Libraries were generated at D0, D3, and D5.

(B) ATAC-seq experimental design.

(C) RNA-seq heatmap for the $Rora^{Low}$ was partitioned into six data-driven clusters (LR1-LR6) across the time course. For each cluster, genes were classified into two primary temporal groups: genes persistently upregulated in $Rora^{Low}$ and genes persistently upregulated in controls. These groups were further categorized by intersecting with ESC and MEF DEG lists derived from pairwise comparisons (defined as fold change ≥ 2; ESC-upregulated, MEF-upregulated, or non-differentially expressed genes).

(D) RNA-seq temporal heatmap analysis of $Rora^{High}$ cells partitioned into six analogous expression clusters (HR1-HR6). Classification followed the same methodology as in panel C, where genes were divided into $Rora^{High}$ upregulated and control-upregulated groups, then categorized by ESC/MEF expression biases. Clusters adhere to the same classification logic and biological definitions established in panel C.

(E) Venn diagram showing the overlap of Day0+Day3+Day5 CUT&Tag peaks among $Rora^{Low}$, $Rora^{High}$, and $Rora^{\Delta NTD}$. Left, Gene Ontology (GO) Biological Process enrichment of genes linked to the shared peak set common to all three conditions (n = 626). Right, GO enrichment of genes linked to the $Rora^{High}$ only peak set (n = 1121). Bars indicate -log10(P value) from Fisher's exact test; numbers in parentheses denote gene counts.

(F) Integration of $Rora^{High}$ only CUT&Tag and High-specific transcriptional programs. The center circle denotes genes linked to $Rora^{High}$ only CUT&Tag peaks, corresponding to the 1,121 High-only peaks in panel E. The left and right circles indicate High-specific downregulated (DC13-DC15; Figure 2D) and upregulated (UC13-UC15; Figure 2C) RNA-seq modules, respectively.

(G) GO Biological Process enrichment of the 50-gene overlap set in panel F.

Figure S5

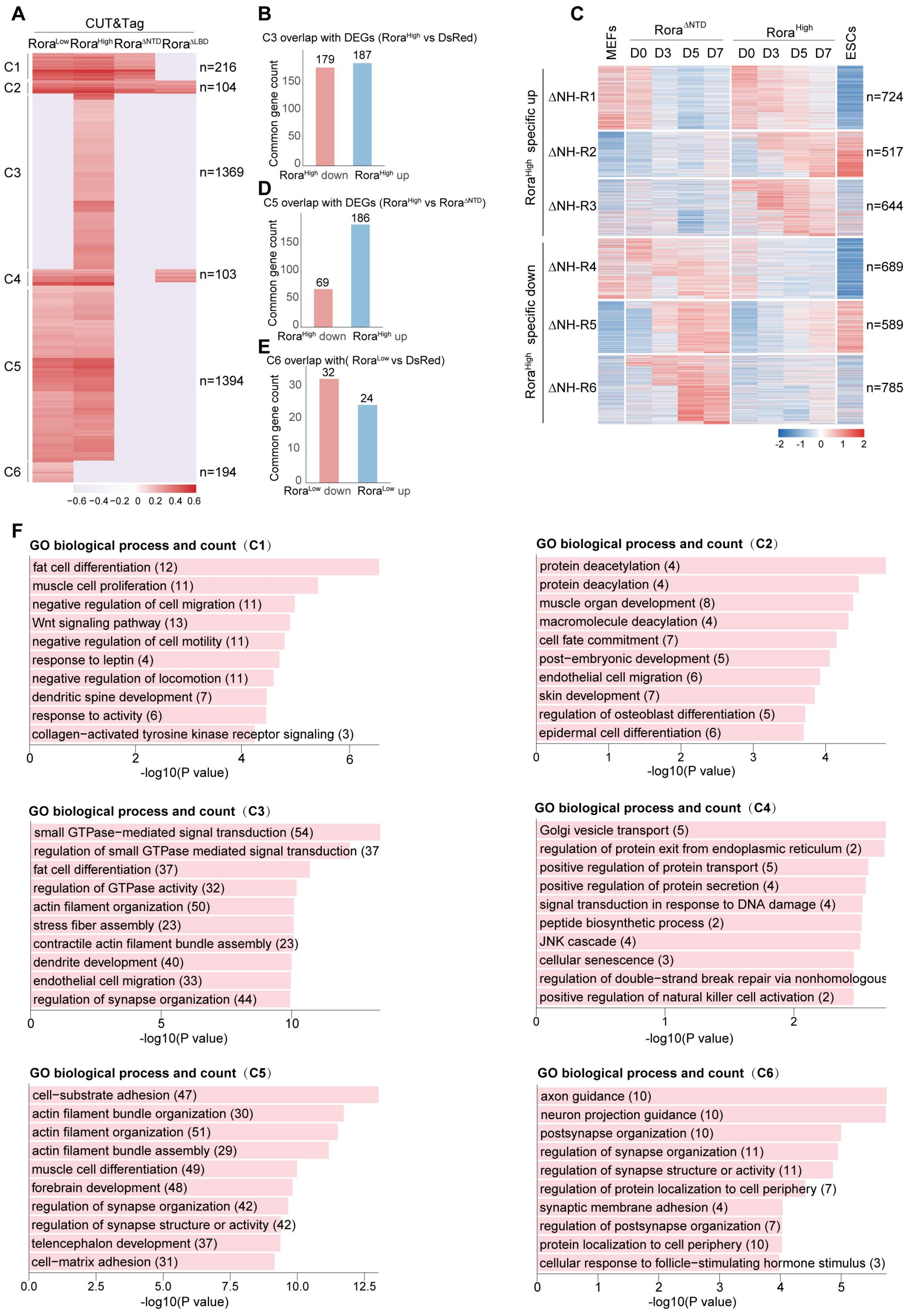

Figure S5. Condition-resolved clustering of RORA CUT&Tag occupancy and linkage to transcriptional outputs. Related to Figure 4.

(A) Heatmap of RORA CUT&Tag signal at Day 0 across the indicated conditions (Rora^{Low}, Rora^{High}, Rora$^{\Delta NTD}$, and Rora$^{\Delta LBD}$). To enable direct comparison of occupancy patterns, peak sets from all conditions were merged into a unified background peak universe using BEDTools, and per-sample pileup signal was log-transformed and hierarchically clustered. Unsupervised

clustering identified six occupancy modules (C1-C6) representing shared and condition-skewed binding patterns; the number of peaks in each module is indicated (right).

(B, D, E) Integration of CUT&Tag modules with RNA-seq outputs. Peaks from the indicated modules were annotated to the nearest genes, and overlap was evaluated with differentially expressed genes (DEGs) from the corresponding RNA-seq contrasts (Figure S4D for $Rora^{High}$ vs DsRed; this figure panel C for $Rora^{High}$ vs $Rora^{\Delta NTD}$; Figure S4C for $Rora^{Low}$ vs DsRed). Bar plots report the number of common genes showing concordant regulation for $Rora^{High}$ vs DsRed (B), $Rora^{High}$ vs $Rora^{\Delta NTD}$ (D), and $Rora^{Low}$ vs DsRed (E), separated into downregulated and upregulated DEGs as indicated.

(C) RNA-seq heatmap of genes corresponding to the CUT&Tag-linked gene sets used in (D), shown across the reprogramming time course for $Rora^{\Delta NTD}$ and $Rora^{High}$. For each cluster, genes were classified into two primary temporal groups: genes persistently upregulated in $Rora^{High}$ and genes persistently upregulated in $Rora^{\Delta NTD}$. These groups were further categorized by intersecting with ESC and MEF DEG lists derived from pairwise comparisons (defined as fold change ≥ 2; ESC-upregulated, MEF-upregulated, or non-differentially expressed genes).

(F) GO biological process enrichment for genes linked to each CUT&Tag occupancy module (C1-C6) from (A). Bars show -log10(*P* value) from Fisher's exact test.

Figure S6

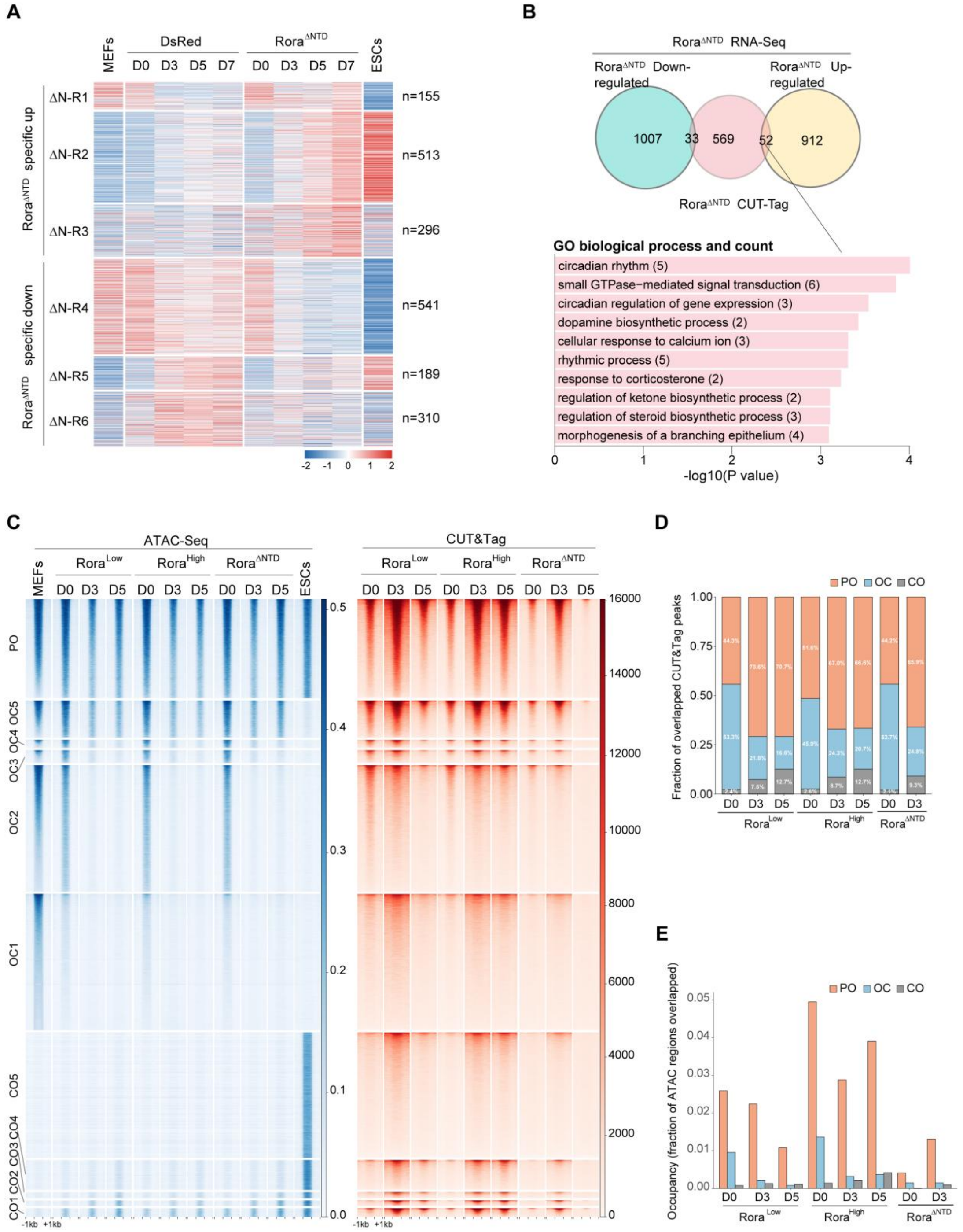

Figure S6. Rora$^{\Delta NTD}$ transcriptome/occupancy features and multi-condition ATAC-CUT&Tag integration. Related to Figure 4.

(A) RNA-seq heatmap for the Rora$^{\Delta NTD}$ was partitioned into six data-driven clusters (ΔN-R1-ΔN-R6) across the time course. For each cluster, genes were classified into two primary temporal groups: genes persistently upregulated in Rora$^{\Delta NTD}$ and genes persistently upregulated in controls. These groups were further categorized by intersecting with ESC and MEF DEG lists derived from pairwise comparisons (defined as fold change ≥ 2; ESC-upregulated, MEF-upregulated, or non-differentially expressed genes).

(B) Integration of Rora$^{\Delta NTD}$ CUT&Tag occupancy with Rora$^{\Delta NTD}$ regulated RNA-seq modules. The center circle denotes genes linked to Rora$^{\Delta NTD}$ CUT&Tag peaks aggregated across CUT&Tag time points D0, D3, and D5 (nearest/peak-associated genes). Left and right circles denote Rora$^{\Delta NTD}$ specific downregulated and Rora$^{\Delta NTD}$ specific upregulated gene modules, respectively, derived from the RNA-seq clustering in panel A. Numbers indicate overlap sizes. The bar plot shows GO Biological Process enrichment for the overlapping gene set highlighted in panel B.

(C) Heatmaps showing ATAC-seq accessibility modules (left; OC/CO/PO defined in Figure 4A) and the corresponding distribution of RORA CUT&Tag signal (right) across Rora^{Low}, Rora^{High}, and Rora$^{\Delta NTD}$ at D0, D3, and D5, enabling direct comparison of RORA occupancy with dynamic chromatin accessibility states.

(D) Composition of RORA CUT&Tag peaks across ATAC accessibility classes. For each condition and time point (Rora^{Low}, Rora^{High}, Rora$^{\Delta NTD}$ at D0/D3/D5), all called RORA CUT&Tag peaks were intersected with ATAC module annotations (PO, OC, CO; defined in Figure 4A). Stacked bars showed the percentage of CUT&Tag peaks falling into PO/OC/CO within each condition-time point, normalized to 100% (i.e., distribution of CUT&Tag peaks across ATAC classes).

(E) ATAC-class-normalized occupancy of RORA binding. For each condition and time point, we quantified RORA binding occupancy for each ATAC class as: (# CUT&Tag peaks overlapping PO/OC/CO) / (# ATAC peaks in PO/OC/CO), thus normalizing CUT&Tag peak counts by the size of the corresponding ATAC module. Bars depict the fraction of ATAC regions in each class that are overlapped by RORA CUT&Tag peaks.

Table S5. Key resources

REAGENT or RESOURCE	SOURCE	IDENTIFIER
Antibodies		
Mouse monoclonal anti-FLAG antibody WB 1:5,000	Sigma-Aldrich	Cat# F1804; RRID: AB_262044
HRP-conjugated Monoclonal Mouse anti-GAPDH WB 1:5,000	KangChen Bio-tech	Cat# KC-5G5; RRID: AB_263120
APC anti-DYKDDDDK Antibody	BioLegend	Cat# 637307
Alexa Fluor 488 anti-SOX2 Antibody	BioLegend	Cat# 656109
Bacterial and virus strains		
DH5α Competent cell	Vazyme Biotech	Cat# C502-02
Chemicals		
Basic DMEM, High Glucose	HyClone	Cat# SH30022.01
Fetal Bovine Serum	NTC	Cat# SFBE
TRYPSIN 0.25% EDTA	Gibco	Cat# 25200114
CHIR-99021	Selleckchem	Cat# S1263
bFGF	PeproTech	Cat# 100-18B
GlutaMAX	Gibco	Cat# 35050079
NEAA	Gibco	Cat# 11140076
SODIUM PYRUVATE SOL	Gibco	Cat# 11360070
beta-mercaptoethanol	Sigma-Aldrich	Cat# M6250
N2	Gibco	Cat# 17502048
B27	Gibco	Cat# 17504044
Vitamin C	Sigma-Aldrich	Cat# 49752
LiCL	Sigma-Aldrich	Cat# L4408
mLIF	LOFETECH	Cat# L00100
Gelatin	Sigma-Aldrich	Cat# G7041
Trizol	MRC	Cat# TR118
VAHTS DNA Clean Beads	Vazyme Biotech	Cat# N411-02
Puromycin Dihydrochloride	Thermo Fisher Scientific	Cat# A1113803
EDTA-free proteinase inhibitor Cocktail	Roche	Cat# 4693132001
Ammonium persulfate (APS)	VWR Chemicals	Cat# VWRC0486
PVDF Membrane	Millipore	Cat# WBKL S0500
DNase/RNase Free Deionized Water	TIANGEN	Cat# RT121
Opti-MEM I	Gibco	Cat# 31985088
Polybrene	Sigma-Aldrich	Cat# H9268
non-fat dry milk	Sangon	Cat# NB0669
PBS	HyClone	Cat# SH30028.02
DPBS	Gibco	Cat# EH80028
Polyethylenimine, PEI	Polysciences	Cat# 24765
Critical commercial assays		
ChamQ SYBR qPCR Master Mix	Vazyme Biotech	Cat# Q311-02
HiScript III RT SuperMix	Vazyme Biotech	Cat# R222-01

ClonExpress II One Step Cloning Kit	Vazyme Biotech	Cat# C112-01
SDS-PAGE Gel Quick Preparation Kit	Beyotime	Cat# P0012AC
Deposited data		
Mass spectrometric data of Rora	This paper	PXD068806
RNA-seq data, CUT&Tag data and ATAC-seq data	This paper	CRA030540
Experimental models: Cell lines		
Platinum-E (Plat-E)	A gift from The Fourth Military Medical University	N/A
Primary mouse embryonic fibroblast	This study	N/A
Software and Algorithms		
ZEN	Zeiss	https://www.zeiss.com/microscopy/int/software cameras.html
Prism 9	GraphPad	https://www.graphpad.com/scientific-software/prism/
Primer used in this paper		
q-mGAPDH-F	AACTTTGGCATTGTGGAAGGGCTCA	
q-mGAPDH-R	TTGGCAGCACCAGTGGATGCAGGGA	
q-m Ifng-F	AGGAACTGGCAAAAGGATGGT	
q-m Ifng-R	ATGTTGTTGCTGATGGCCTG	
q-Exo-Oct4-F	GGGTGGACCATCCTCTAGAC	
q-Exo-Oct4-R	CCAGGTTCGAGAATCCAC	
q-Exo-Sox2-F	GGGTGGACCATCCTCTAGAC	
q-Exo-Sox2-R	CTTCAGCTCCGTCTCCATCA	
q-Exo-Klf4-F	GGGTGGACCATCCTCTAGAC	
q-Exo-Klf4-R	GCTGGACGCAGTGTCTTCTC	
q-Exo-Rora-R	ATTCCTGACGATTTGTCTCCAC	
q-VCN-Alb-F	TCCAAACCTCCGTGAAAACTATG	
q-VCN-Alb-R	TGTGTTGCAGGAAACATTCGT	
Recombinant DNA		
PMXs-Oct4	This study	N/A
pMXs-Sox2	This study	N/A
pMXs-Klf4	This study	N/A
pMXs-DsRed	This study	N/A
pMXs-Nr0b1	This study	N/A
pMXs-Nr0b2	This study	N/A
pMXs-Thra	This study	N/A
pMXs-Thrb	This study	N/A
pMXs-Rara	This study	N/A
pMXs-Rarb	This study	N/A
pMXs-Rarg	This study	N/A

pMXs-Ppara	This study	N/A
pMXs-Pparb	This study	N/A
pMXs-Nr1c3	This study	N/A
pMXs-Nr1d1	This study	N/A
pMXs-Nr1d2	This study	N/A
pMXs-Rora	This study	N/A
pMXs-Rorb	This study	N/A
pMXs-Rorc	This study	N/A
pMXs-Nr1h3	This study	N/A
pMXs-Nr1h2	This study	N/A
pMXs-Nr1h4	This study	N/A
pMXs-Nr1h5	This study	N/A
pMXs-Vdr	This study	N/A
pMXs-Nr1i2	This study	N/A
pMXs-Nr1i3	This study	N/A
pMXs-Nr2a1	This study	N/A
pMXs-Hnf4g	This study	N/A
pMXs-Nr2b1	This study	N/A
pMXs-Nr2b2	This study	N/A
pMXs-Rxrg	This study	N/A
pMXs-Nr2c1	This study	N/A
pMXs-Nr2c2	This study	N/A
pMXs-Nr2e1	This study	N/A
pMXs-Nr2e3	This study	N/A
pMXs-Nr2f1	This study	N/A
pMXs-Nr2f2	This study	N/A
pMXs-Nr2f6	This study	N/A
pMXs-Esr1	This study	N/A
pMXs-Esr2	This study	N/A
pMXs-Esrra	This study	N/A
pMXs-Esrrb	This study	N/A
pMXs-Nr3b3	This study	N/A
pMXs-Nr3c1	This study	N/A
pMXs-Nr3c2	This study	N/A
pMXs-Nr3c3	This study	N/A
pMXs-Nr3c4	This study	N/A
pMXs-Nr4a1	This study	N/A
pMXs-Nr4a2	This study	N/A
pMXs-Nr4a3	This study	N/A
pMXs-Nr5a1	This study	N/A
pMXs-Nr5a2	This study	N/A
pMXs-Nr6a1	This study	N/A

Supplemental methods

Flow cytometry

Cells were dissociated into single-cell suspensions, washed in PBS, and fixed/permeabilized using a commercial fixation/permeabilization kit following the manufacturer's instructions. Cells were stained with anti-FLAG-APC and anti-SOX2-FITC for 30 min at room temperature in the dark, washed, and analyzed on a flow cytometer. Compensation was performed using single-stained controls, and data were analyzed in FlowJo.

Viral genome copies quantification

Viral genome copies in viral supernatants were quantified using a Takara qPCR-based viral titration kit according to the manufacturer's protocol. Ct values were converted to copies/mL using the kit-provided standards and standard curve. All reactions were run with technical replicates and included no-template controls.

Vector copy number (VCN) assay

Vector copy number (VCN) was estimated by qPCR on genomic DNA using primers targeting a vector-specific sequence and a single-copy reference gene (Alb). Ct values were used to compute relative VCN as 2^ (Ct_reference − Ct_vector) (ΔCt method). All reactions were run in technical replicates and included no-template controls. Relative VCN values were compared across conditions using equal gDNA input per reaction.

Quantitative RT-PCR (qRT-PCR)

Total RNA was isolated using the Trizol method. For quantitative PCR analysis, cDNA was first synthesized from the extracted RNA using Hiscript II Q RT SuperMix for qPCR. Subsequent qPCR reactions were then performed using ChamQ SYBR qPCR Master Mix on a real-time PCR detection system. Each sample was assayed in three technical replicates. *Gapdh* was used as the internal reference gene, and the relative gene expression was calculated using the $2^{(-\Delta\Delta Ct)}$ method. The primer sequences used for quantitative PCR are listed in Table S5.

Western blotting

Western blotting was performed using standard laboratory protocols, with gels primarily prepared using the Beyotime P0012Ac kit.

Cells were washed twice with cold PBS and lysed in a buffer containing 50 mM Tris-HCl (pH 6.8), 80 mM NaCl, 2% SDS, 0.4% NP-40, 0.8 mM EDTA, 10% glycerol, 1% β-mercaptoethanol, 0.02% bromophenol blue, and a protease inhibitor cocktail. After boiling for 10 min, samples were centrifuged at 13,000 × g, and the supernatants were subjected to SDS-PAGE followed

by transfer to polyvinylidene difluoride (PVDF) membranes.

Membranes were blocked with 5% (w/v) non-fat dry milk in TBST (10 mM Tris-HCl, pH 8.0, 150 mM NaCl, 0.05% Tween-20) for 1 h at room temperature. After three 10-min washes with TBST, membranes were incubated with primary antibodies overnight at 4°C. Following another three TBST washes, membranes were probed with appropriate secondary antibodies for 1 h at room temperature. GAPDH served as the loading control. All Western blot analyses were performed with at least two biological replicates, and consistent results were observed. Antibodies used are listed in Table S5 **.**

RNA-seq

For RNA sequencing, total RNA was extracted as described above. Using 1 μg of total RNA per sample, libraries were constructed with the VAHTS™ Total RNA-seq (H/M/R) Library Prep Kit for Illumina® (Vazyme Biotech). The procedure was as follows: ribosomal RNA was first removed using the Ribo-off Depletion Kit, followed by purification of the ribosomal-depleted RNA with mRNA Capture Beads and fragmentation at 94 °C for 8 minutes using Frag/Prime Buffer. Subsequently, double-stranded cDNA was synthesized, adapters were ligated, and the fragments were purified with DNA clean beads. After PCR amplification for 15 cycles, the final RNA libraries were obtained. All libraries were sequenced on an Illumina NovaSeq 6000 system.

Cut&Tag

We performed CUT&Tag sequencing on six sample groups at Day 0 (D0), Day 3 (D3), and Day 5 (D5) using the Hyperactive Universal CUT&Tag Assay Kit for Illumina (Vazyme Biotech, TD903), strictly following the manufacturer's protocol. The procedure is briefly outlined as follows: For each sample, 100,000 cells were collected, washed, and bound to pre-activated ConA magnetic beads for 10 minutes at room temperature. Subsequently, diluted Anti-FLAG primary antibody (Sigma, #F1804) was added and incubated overnight at 2-8°C, followed by incubation with a species-matched secondary antibody for 30-60 minutes at room temperature. Washes were performed using Dig-Wash Buffer between incubation steps. Next, pA/G-Tn5 transposase was added and reacted at room temperature for 1 hour. After washing, fragmentation was conducted with TTBL Buffer at 37°C for 60 minutes. Finally, Proteinase K digestion was performed, DNA was extracted using magnetic beads, and libraries were constructed via PCR amplification. After passing quality control, sequencing was carried out on the Illumina NovaSeq 6000 platform.

ATAC-seq

This study performed ATAC-seq sequencing on six sample groups at four time points (D0, D3, D5 and D7), strictly following the standardized protocol of the Hyperactive ATAC-seq Library Kit for Illumina (Vazyme Biotech, TD711). The key steps included: nuclei preparation and

transposition reaction, where cells were collected, washed with pre-cooled TW Buffer, and lysed with Lysis Buffer to release nuclei, followed by the addition of a pre-configured fragmentation Mix for a 30-minute transposition reaction in a 37°C water bath to simultaneously fragment chromatin and ligate sequencing adapters, terminated with Stop Buffer; DNA purification and library construction, where DNA fragments from the transposition product were purified using the ATAC DNA Extract Beads and directly subjected to PCR amplification for library construction; and finally, library purification and size selection, where ATAC DNA Clean Beads were used in a two-step purification process to isolate DNA fragments of appropriate length, ultimately yielding high-quality sequencing libraries.

IP-MS

MEFs overexpressing DsRed, WT-*Rora* and *Rora* mutants were lysed using lysis buffer (containing protease inhibitors). Subsequently, 1 mg of nuclear lysate was incubated with Flag-M2 magnetic beads at 4°C for 1.5 hours for co-immunoprecipitation. After washing with lysis buffer and PBS, on-bead digestion was performed as follows: sequential treatment with elution buffer containing DTT and urea, iodoacetamide (for alkylation), and trypsin (for partial digestion). The eluates were combined and further digested with trypsin overnight at room temperature. The resulting peptides were acidified with trifluoroacetic acid and desalted using Thermo Scientific Acclaim PepMap 100 C18 column with a 140-minute gradient elution (Buffer B: 80% acetonitrile, 0.1% formic acid) for separation, followed by column washing; the analysis was performed on an Thermo Scientific Easy nLC 1200 system coupled online to a Thermo Fisher Fusion Lumos mass spectrometer; and data were acquired in data-dependent acquisition mode with a 90-second dynamic exclusion setting. All experiments were performed with three biological replicates.
